# Emerging Strategies in Enhancing Singlet Oxygen Generation of Nano-Photosensitizers Toward Advanced Phototherapy

**DOI:** 10.1007/s40820-022-00856-y

**Published:** 2022-05-05

**Authors:** Mohammad Tavakkoli Yaraki, Bin Liu, Yen Nee Tan

**Affiliations:** 1 Institute of Materials Research and Engineering, The Agency for Science, Technology and Research (A*STAR), 2 Fusionopolis Way, #08-03, Innovis, 138634 Singapore; 2 Department of Chemical and Biomolecular Engineering, National University of Singapore, 4 Engineering Drive 4, Singapore, 117585 Singapore; 3 Faculty of Science, Agriculture and Engineering, Newcastle University, Newcastle Upon Tyne, NE1 7RU UK; 4 Newcastle Research and Innovation Institute, Newcastle University in Singapore, 80 Jurong East Street 21, #05-04, Singapore, 609607 Singapore

**Keywords:** Nano-photosensitizer, Reactive oxygen species, Aggregation-induced emission, Metal nanocluster, Carbon dots, Metal-enhanced singlet oxygen generation

## Abstract

The great promise of photodynamic therapy (PDT) has thrusted the rapid progress of developing highly effective photosensitizers (PS) in killing cancerous cells and bacteria. To mitigate the intrinsic limitations of the classical molecular photosensitizers, researchers have been looking into designing new generation of nanomaterial-based photosensitizers (nano-photosensitizers) with better photostability and higher singlet oxygen generation (SOG) efficiency, and ways of enhancing the performance of existing photosensitizers. In this paper, we review the recent development of nano-photosensitizers and nanoplasmonic strategies to enhance the SOG efficiency for better PDT performance. Firstly, we explain the mechanism of reactive oxygen species generation by classical photosensitizers, followed by a brief discussion on the commercially available photosensitizers and their limitations in PDT. We then introduce three types of new generation nano-photosensitizers that can effectively produce singlet oxygen molecules under visible light illumination, i.e., aggregation-induced emission nanodots, metal nanoclusters (< 2 nm), and carbon dots. Different design approaches to synthesize these nano-photosensitizers were also discussed. To further enhance the SOG rate of nano-photosensitizers, plasmonic strategies on using different types of metal nanoparticles in both colloidal and planar metal-PS systems are reviewed. The key parameters that determine the metal-enhanced SOG (ME-SOG) efficiency and their underlined enhancement mechanism are discussed. Lastly, we highlight the future prospects of these nanoengineering strategies, and discuss how the future development in nanobiotechnology and theoretical simulation could accelerate the design of new photosensitizers and ME-SOG systems for highly effective image-guided photodynamic therapy.

## Highlights

- Recent advancement in Type II nano-photosensitizers (AIE nanodots, carbon dots and metal nanoclusters) are reviewed.
- Nanoplasmonic strategies in enhancing singlet oxygen generation efficiency of different metal-photosensitizer (planar and colloidal) systems are discussed.
- Current challenges and future prospects of metal-enhanced nano-photosensitizers for advanced photodynamic therapy and theranostic treatment are highlighted.

## Graphical abstract

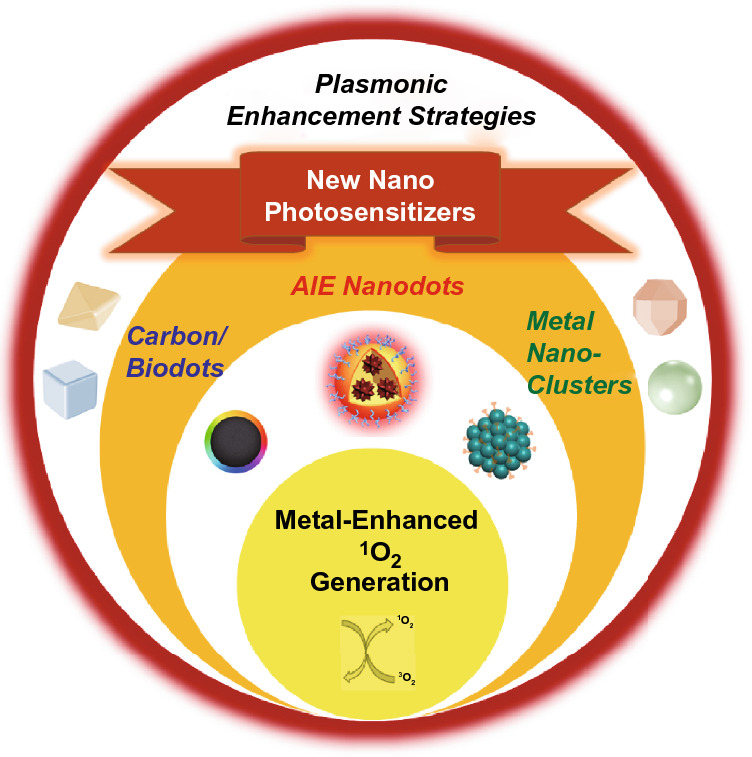

## Introduction

Light plays a vital role in the whole human society as well as science and technology. Seeking deep understanding of light-mediated processes is of interest, especially in nanomedicine, as light not only enables us to visualize different processes and species that have never seen before [1–3], but also initiates some important reactions in the biological systems, e.g., photosynthesis [4]. One of such exploitations of light in nanomedicine is the implementation of photodynamic therapy (PDT) which earned the Noble Prize in Medicine and Physiology for Niels Ryberg Finsen in 1903. PDT is a non-invasive technique that could be used for treatment of different diseases including cancers. It can also be used in combination with other treatment methods to enhance the effectiveness of the whole therapy. Photosensitizer is the key component in PDT, which can be used to generate reactive oxygen species (ROS), when they are irradiated by light. In general, there are two types of photosensitizers based on the types of reactive oxygen species being generated. Type I photosensitizer transfers electron to the surrounding biological molecules, yielding free radicals. These radicals can interact with oxygen and water molecules to produce hydroxyl and superoxide anions. On the hand, Type II photosensitizer transfers energy to the surrounding oxygen molecules and produces singlet oxygen molecules. During light irradiation, the excited electrons of Type II photosensitizers undergo intersystem crossing (ISC) process and then transfer their energy to the surrounding oxygen species to generate singlet oxygen. The as-generated singlet oxygen molecules could interact with the cancerous cells, causing damage to their membrane or essential constituent proteins, leading to the cell death. Among different types of ROS, single oxygen has been widely studied for PDT. PDT is advantageous over other therapeutic treatments such as chemotherapy owing to its biocompatibility and ability to control the dosage with light (e.g., power and irradiation time) [5–7].

The great promise of photodynamic therapy has thrusted the rapid progress of developing photosensitizers from the use of hematoporphyrin and photofrin to the second generation of photosensitizers that are based on porphyrin and non-porphyrin molecules. However, most of these photosensitizers have large planar structures, which suffer from the aggregation-caused quenching (ACQ), leading to weak fluorescence and poor ROS generation efficiencies [8]. The recent advancements in nanotechnologies have led to the development of nanomaterials-based photosensitizers (also known as nano-photosensitizers) with enhanced photostability and singlet oxygen generation (SOG) efficiency. Different type of nanomaterials with SOG properties such as aggregation-induced emissive (AIE) nanodots, carbon-based quantum dots, upconversion nanoparticles, and ultrasmall metal nanoclusters can be molecularly designed and engineered to possess unique physiochemical properties such as photosensitization and photoluminescence that can overcome most of the limitations of traditional photosensitizers [9–16]. In addition, noble metal nanomaterials such as gold and silver nanoparticles can be used to enhance the SOG efficiency of photosensitizers through plasmonic coupling. This approach relies on the interactions of light with the plasmonic nanoparticles and photosensitizer that are placed in proximity. This plasmon-enhancement strategy can be applied to accelerate the rate of SOG for different types of photosensitizers [17–22]. These metal-enhanced photosensitizers often show higher photostability and minimum photobleaching, rendering it an excellent theranostic agent for image-guided therapy [20–24].

In this paper, we focus on the review of recently developed nano-engineered photosensitizers (Type II) and the emerging strategies such as plasmonic engineering that lead to effective singlet oxygen generation toward advanced phototherapy, which have not been covered in the recently published review papers [25–28]. We first explain the general mechanism of ROS generation by the photosensitizer including Type I and II PSs, followed by brief discussion on a few commercially available photosensitizers and their limitations in PDT. We then introduce three different new generation nano-photosensitizers that can effectively produce singlet oxygen molecules under visible light illumination, i.e., aggregation-induced emission (AIE) nanodots, metal nanoclusters (< 2 nm), and carbon dots. The photophysical and photochemical properties of these nano-photosensitizers such as photostability, fluorescent brightness, SOG efficiency as well as their applications in PDT are summarized. In addition, the emerging strategies for SOG enhancement by using different types of photosensitizers and plasmonic nanoparticles in both planar and colloidal systems are reviewed. The key parameters that determine the SOG efficiency and their underlined enhancement mechanism are discussed. Lastly, the future development and prospects of nano-photosensitizers in PDT are highlighted, suggesting the importance of the fundamental knowledge of photo- and nano-chemistry as well as better understanding of the SOG enhancement strategies and biological impacts to their actual applications in nanomedicine.

## Photosensitizer Development: From Classical Molecular Design to Nano-Engineering

Due to the crucial role of a photosensitizer in ROS generation, it is central to develop effective photosensitizers toward the real application of PDT. Over nearly two centuries, thousands of photosensitizers have been developed and many have gone through clinical trials and got the approval as commercial therapeutics for cancer treatment. Yet, most of these commercial photosensitizers still suffer from the issues like low photo-stability, dark cytotoxicity, poor water solubility and inefficient singlet oxygen generation in aggregated states. These drawbacks have greatly impeded the performance of these photosensitizers in PDT. In this section, we briefly introduce the mechanism of ROS generation and outline the key role of the photosensitizer in PDT. Then, we summarize different types of commercial photosensitizers, by providing critics of their limitations that motivate the exploration of new generation of nano-photosensitizers for SOG. They are the (1) AIE molecules-based, (2) metal nanoclusters-based and (3) carbon dots-based nano-photosensitizers, which will be discussed in the following sub-sections.

### Types of Photosensitizers in Reactive Oxygen Species Generation

**Fig. 1:**
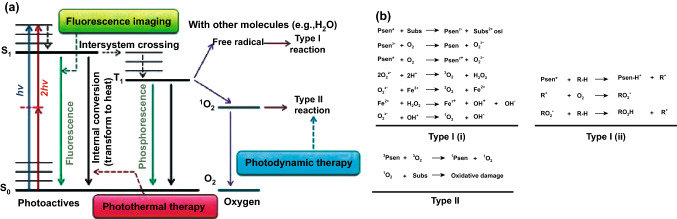
**a** Simplified Jablonski diagram for photosensitizer molecule, reproduced from Ref. [29] with permission from Wiley Online Library. **b** Possible interactions of photosensitizer molecule with surrounding molecules via different types of reactions (Type I and II), reproduced from Ref. [30] with permission from Hindawi

The generation of ROS involves the energy transfer from a photosensitizer to the oxygen molecule upon light irradiation. The whole photosensitization process is complex but can be simplified using the Jablonski diagram as shown in Fig. 1a. When a photosensitizer molecule is irradiated by appropriate incident light, the electron transits from the lowest ground state (*S*_0_) to singlet excited states (*S*_1_, *S*_2_, *S*_3_, etc.). These excited electrons could either go back to the ground states through a pathway that combine internal conversion and vibration relaxation or go to triplet-excited states via intersystem crossing. The former route is a fast process (in the order of 10^–15^ s) and gives off the excess energy in the form of heat, while the latter one is critical for the generation of ROS. As the ISC occurs with a change in the electron spin, it is called the spin-forbidden process and the population of excited electrons in the *T*_1_ is much less than *S*_1_. Fluorescence and phosphorescence could be observed when the excited electrons go back to the ground state via emission of photons from *S*_1_ and *T*_1_ to the ground state (*S*_0_), respectively [29].

As compared to other molecules, photosensitizers have much more population of electrons in the excited triplet states with a longer lifetime (in the orders of a few to hundreds of µs) [31]. These long-lived triplet states enhance the probability of interaction and energy transfer between excited triplet photosensitizer and the surrounding molecules like water and oxygen in the physiological mediums. This energy transfer process involves a series of complicated reactions [32–34], which could be described by the two main pathways, named as type I and type II, respectively, as illustrated in Fig. 1b [30, 35]. Type I reactions include the energy transfer between the excited triplet state of photosensitizers and a substrate. There are two scenarios for type I reactions. In the first Type I (i) scenario, electrons are transferred from the substrate to the excited triplet state of photosensitizers, resulting in the formation of cationic radical substrate and anionic radical photosensitizer molecule. These radicals could react with oxygen molecules in the environment instantaneously and produce a complex mixture of oxygen intermediates (e.g., superoxide). While the Type I (ii) scenario typically involves transferring of a hydrogen atom (reduction) to the excited triplet of photosensitizer molecule. Type I reactions generate diverse radicals leading to the formation of different ROS in the medium. The most common ROS that are generated via the type I pathway are superoxide anion, hydroxyl radical, and/or hydrogen peroxide.

On the other hand, Type II reactions are based on the direct energy transfer between the excited triplet state of photosensitizer molecule and the surrounding oxygen molecules, resulting in SOG via different triplet–triplet annihilation processes. The generated singlet oxygen is the most effective and essential ROS in the PDT that could act in the radius of 20 nm, with approximate lifetime of 40 ns in the biological systems [6, 36]. The singlet oxygen molecule could react with different biological molecules including amino acid residues in proteins (e.g., tryptophan), nucleic acid bases, particularly guanosine and guanine derivatives, and unsaturated lipids like cholesterol where these interactions could result in cell death due to cell membrane damage or protein deactivation in the cells [37, 38]. It should be mentioned that the ROS generation process is more complicated in the biological media. Many factors, including but not limited to absorption cross section of photosensitizers, low rate of ISC process, intracellular oxygen concentration, and energy transfer to other molecules, can affect the actual ROS generation efficiency of a photosensitizer. All these factors should be carefully considered when designing new photosensitizers for successful PDT applications.

### First and Second Generations of Photosensitizers

As the most important component in PDT, the photosensitizer has been extensively studied. Different types of photosensitizers have been synthesized with various notable strategies to enhance the ROS generation rate. For instance, different electron donor and electron acceptor groups have been introduced to control the intersystem crossing rate. In this section, we briefly review the development of different types of photosensitizers and their commercial products from its first discovery to the development of new generation of photosensitizers based on nanomaterials.

#### The first Generation of Photosensitizers

**Fig. 2:**
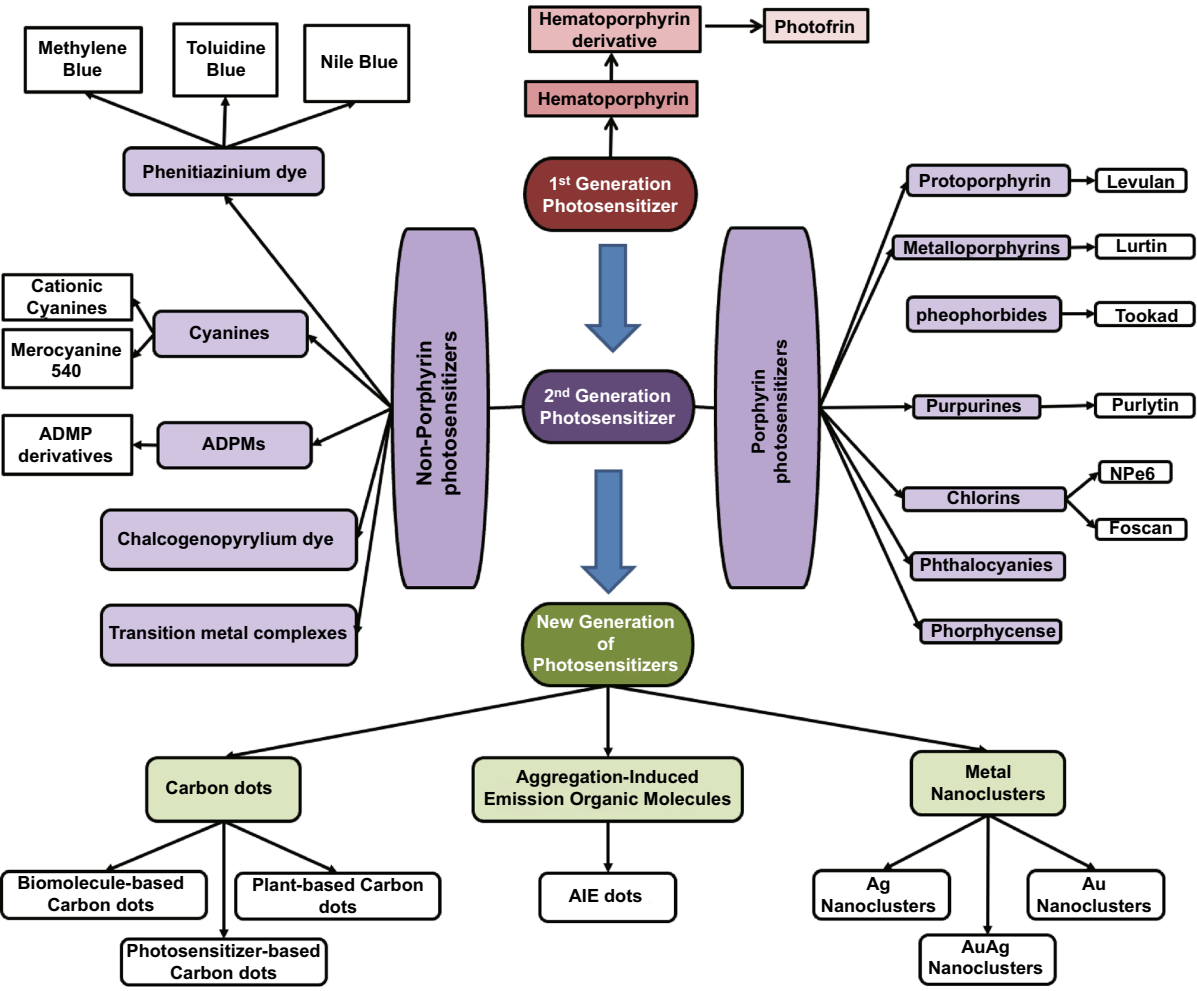
Classification of different generations of photosensitizers including new generation of nano-photosensitizers that generate singlet oxygen as reviewed in this paper

Figure 2 shows the classification of different types of photosensitizers. The first observation of photosensitizing effect can be dated back to 1841 when hematoporphyrin was first discovered when removing the iron from dried blood. Following the next twenty years, many attempts have been made to purify the hematoporphyrin and apply it for diagnosis and therapy purposes. However, a high dosage is often needed for this compound and its accumulation in the targeted tissue is very low, which hinders its wide application. Porphyrin, which is the lyophilized concentrated form of monomeric and oligomeric hematoporphyrin derivatives obtained by partial purification of impure hematoporphyrin, was then introduced for clinical use and is still remains as the gold standard for PDT treatment of non-skin cancers. Both hematoporphyrin and its derivatives were conventionally classified as the first-generation of photosensitizers. They have low light absorption coefficient in the near-infrared wavelength and suffer from weak penetration into tumor tissue. Moreover, the enforcement of patients to stay out of sunlight for at least 4 weeks post-treatment after administration of these photosensitizers (due to side reactions as result of photosensitization effect) further prolongs the treatment process. To overcome these limitations, researchers have been trying to develop alternative photosensitizers, which leads to the development of the second generation of photosensitizers.

#### The Second Generation of Photosensitizers

**Fig. 3:**
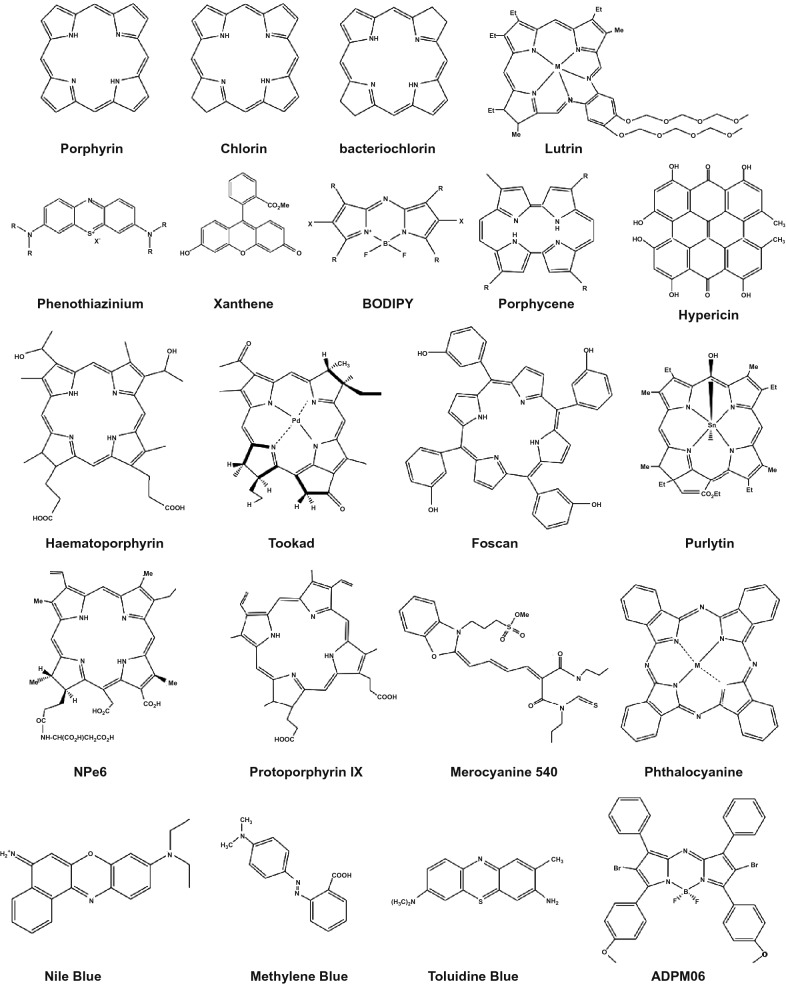
The chemical skeleton of some classical photosensitizers

The second generation of photosensitizers includes those new molecules that have been developed to overcome the limitations of the afore-mentioned first generation of photosensitizers in PDT application in oncology. They can be classified into two main groups according to their molecular framework: (1) Porphyrin-based and (2) non-porphyrin-based photosensitizers (Fig. 2). The chemical skeleton/structure of these photosensitizers is presented in Fig. 3. Porphyrins are a class of macrocycle molecules that are highly conjugated and have central metal atoms (e.g., iron, zinc or magnesium) which absorb intensely at longer wavelengths due to abundance in *π* electrons in their structures. Porphyrins are naturally occurring, which can be found in human body as well. They play an essential role in the biological activities of all living organisms. Note that the discovery of photosensitizing effect was first found in hematoporphyrin, which is also a type of porphyrins. As hematoporphyrin has been struggling for the afore-mentioned limitations, researchers have been looking for strategies to fine-tune the structure of porphyrin in order to enhance their properties. As one of such efforts, side chains with different functional groups such as nitrogen [39], carboxyl [40], sugar [41], ethylenediaminetetraacetic acid [40], silyl [42], and isoquinoline [43], have been introduced to the main skeleton of porphyrin to improve its physicochemical properties as well as singlet oxygen generation quantum efficiency. Moreover, preparation of metal-porphyrin conjugates by incorporation of metal ions like zinc, platinum, indium, and iron into the porphyrin structure is another strategy to improve its singlet oxygen generation efficiency [41, 44].

Chlorins are another class of photosensitizers that are of interest due to their higher near infrared absorbance as compared with porphyrins. However, they suffer from low water solubility. This limitation could be overcome by conjugation with water-soluble groups like amino acids, peptides, and sugars [45]. The PDT efficiency of chlorins can be further improved by introducing heavy atoms like borons to their structure [46]. Other than chlorins, phthalocyanines are also a member of porphyrin-based photosensitizer family that have shown promising PDT effects. However, these molecules also suffer from low water-solubility and quenching of singlet oxygen when they are used at high concentrations due to the *π*–*π* stacking and aggregation [47]. To overcome these limitations, a similar strategy of conjugation with water-soluble moieties like cationic or anionic groups, peptide, crown ethers, *β*-cyclodextrins, etc. has been employed [48–51]. On the hand, paramagnetic and diamagnetic texaphyrin complexes are another subclass of porphyrin-based photosensitizer. Texaphyrins are similar to porphyrins in structure but vary in the number of *π* electrons and number of planar nitrogen atoms, which causes a red shift in the absorbance as compared to porphyrins and makes them more suitable for PDT application due to their excitation wavelengths.

Although porphyrin-based photosensitizers are efficient in singlet oxygen generation, they generally suffer from low water-solubility. Modifications must be done to their structure to improve their water solubility. However, no clear design rules have been established yet. In view of this issue, researchers have been seeking after non-porphyrin-based photosensitizers with high ROS generation efficiencies and higher water-solubility, which constituent the majority of so-called second generation of photosensitizers, including phenothiazinium dyes, cyanines, ADPMs, and chalcogenopyrylium dye, to name a few. Phenothiazinium dyes include methylene blue [52], Nile blue [53] and toluidine blue [54] and their analogs (Fig. 2). These dyes can produce singlet oxygen with high efficiency and have been used for different PDT applications. Cyanines and its derivatives are also promising PDT agents [55]. Besides, some complexes of transition metal ions such as Ru(II), Os(II), and Ir(III) have shown efficient singlet oxygen generation with NIR absorbance maximum peak. Most of these complexes were based on bipyridine, bipyrazine, and 2,2′-bipyrimidine where Ru(II)-based complexes were more efficient [56–58].

Table 1 summarizes the different photosensitizer molecules and their singlet oxygen quantum efficiencies (*ϕ*Δ). It was found that the type of metal ion in these complexes could directly affect the fluorescence lifetime as well as singlet oxygen quantum efficiency [59, 60]. However, these dyes are still far from ideal for clinical applications due to certain limitations, such as less selective accumulation in targeted tissue, quenching PDT efficiency in the aggregated state, dark toxicity, rapid excretion, and metabolic inactivation. It is still in pressing need to develop new (other than first and second generations of photosensitizers as listed here) with more favorable photophysical properties for PDT applications [55, 61].

**Table 1:** Different photosensitizers and their singlet oxygen generation quantum efficiencies (***ϕ***Δ) as-obtained from the literature [52–60]

Type II photosensitizer	***ϕ***Δ (D_2_O)	***ϕ***Δ (H_2_O)	***ϕ***Δ (CH_3_CH_2_OH)	***ϕ***Δ (CH_3_OH)	***ϕ***Δ (C_6_H_6_)	***ϕ***Δ (CH_3_OD)
Rose Bengal	0.76	0.75	0.68	0.76	–	–
Fluorescein	–	0.03	0.03	0.1	–	–
Eosin blue	–	0.52	0.37	–	–	–
Methylene blue	–	–	0.52	0.5	–	–
Erythrosin blue	–	0.63	0.69	–	–	–
H_2_TPP	–	–	–	–	0.63	–
MgTPP	–	–	–	–	0.62	–
ZnTPP	–	–	–	–	0.83	–
PdTPP	–	–	–	–	0.88	–
Pc	–	–	–	–	–	0.16
PcTS^4−^	–	–	–	–	–	0.17
ZnPcTS	–	–	–	–	–	0.45
Cd(Tex)(OAc)	–	0.24	–	–	–	–
Y(Tex)(OAc)_2_	–	0.58	–	–	–	–
In(Tex)(OAc)_2_	–	0.48	–	–	–	–
Lu(Tex)(OAc)_2_	–	0.31	–	–	–	–
Eu(Tex)(OAc)_2_	–	0.091	–	–	–	–
Gd(Tex)(Oac)_2_	–	0.08	–	–	–	–

### New Generation of Nano-Photosensitizers

Recent years, nanomaterials with photosensitization properties have been developed with an aim to overcome the drawbacks of classical photosensitizers, such as limited water solubility, poor photostability, and low ROS generation due to ACQ effect. In this session, we review three types of nano-photosensitizers that generate single oxygen with unique photochemical and photodynamic properties with good biocompatibility due to their ultrasmall size and the use of organic molecules and/or biomolecules for the synthesis. Additionally, owing to the versatility of surface chemistry on these three types of nano-photosensitizers, they could be further functionalized with the biorecognition elements (e.g., antibody and aptamer) through simple chemical modification, enabling targeted photodynamic therapy of diseases cells, tissues or tumors. The first one is AIE nanodots [20–22]. AIE molecules are a new class of organic molecules that are non-emissive in dispersed form but strongly luminesce upon aggregation [62–64]. In contrast to the common fluorophores-based photosensitizers, their light-harvesting and ROS generation properties are not affected by the ACQ effect upon aggregation. Here, we focus on the Type II AIE nanodots that generate singlet oxygen only. The second type is the ultrasmall biotemplated metal nanoclusters (NC) [12, 65–71] with core size less than 2 nm (or equivalently 150 metal atoms) that are protected by biomolecular shell. They possess both bright fluorescence emission and singlet oxygen generation ability. The last type is the carbon-based nano-photosensitizer derived from biomolecular precursors (also known as biodots) that exhibit strong photoluminescence and photosensitization properties, particularly singlet oxygen generation In addition, they are inherently biocompatible due to their biomolecular derived origin. The recent progresses of these three types of nano-photosensitizers in SOG and photodynamic therapy applications are highlighted in the following sections.

#### AIE Nanodots-Based Photosensitizers (Type II)

A practical concern of PDT treatment is its long-lasting potency, which can be addressed by applying high loading capacity of photosensitizer molecules or photosensitizer molecules with high photostability throughout the course of light irradiation. A high loading capacity may also increase the dark toxicity of photosensitizer molecules and thus the latter approach might be more favorable. However, traditional organic dye-based photosensitizers are suffering from decomposition under prolonged light irradiation due to their poor photostability. Worse still, their SOG generation properties are greatly hampered by aggregation caused quenching due to the hydrophobic nature of these PS molecules. Hence, designing new PS molecules which retain its SOG generation ability in the aggregated state and are highly stable against photobleaching is of practical importance.

**Fig. 4:**
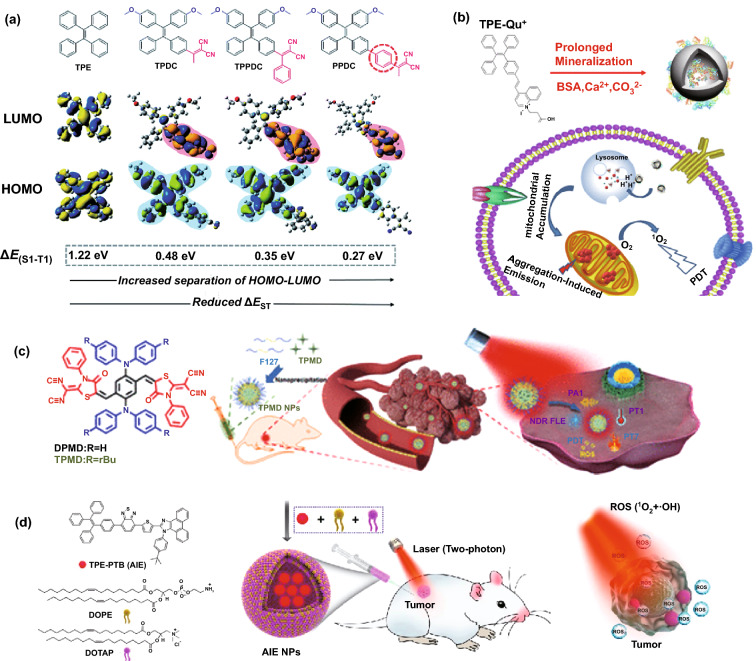
**a** Chemical structures and HOMO–LUMO distributions of AIE photosensitizers designed by introducing different donor and acceptor groups to the TPE molecule and its effect on Δ*E*_ST_, reproduced from Ref. [11] with permission from the Royal Society of Chemistry. **b** Molecular structure of TPE-Qu^+^ AIE photosensitizer, its nanoparticle formation via mineralization, and decomposition of TPE-Qu^+^-loaded CaCO_3_ nanoparticles inside the target cell, reproduced from Ref. [88] with permission from American Chemical Society. **c** Molecular structure of TPMD AIE photosensitizer and its encapsulation into nanoparticle using P127 for PDT and PTT applications, Reproduced from Ref. [89] with permission from American Chemical Society. **d** Design and Preparation of lipid-encapsulated TPE-PTB-based AIE nanoparticles for two-photon fluorescence imaging-guided photodynamic therapy application, reproduced from Ref. [91] with permission from American Chemical Society

AIE dyes could solve the ACQ problem faced by conventional organic dye photosensitizers [72–75]. Typically, they contain a tetraphenylethene, tetraphenylsilole, or triphenylamine moiety which is the active component making the AIE molecules to light up upon aggregation [76–82]. To suit for various applications, additional groups can be introduced to the backbone of AIE dyes in enhancing their photostability with better light-harvesting properties and brighter emissions. Currently, there are two approaches for the design of Type II AIE-based photosensitizers. The first approach is to synthesize the AIE molecule with small singlet–triplet energy gap (Δ*E*_ST_) in order to have the high probability of intersystem crossing rate and thus a higher chance of sensitizing surrounding oxygen molecules to produce singlet oxygen. Since the backbones of AIE molecules have wide Δ*E*_ST_ (e.g., Δ*E*_ST_ for tetraphenylethene is 1.22 eV), they cannot be readily applied for PDT (Fig. 4a). This can be solved by introducing different donor and acceptor groups to the backbone AIE molecules to narrow the singlet–triplet energy gap and improve their SOG generation ability [11, 83–85]. For example, Gu et al. [86] have introduced methoxy group as donor and dicyanovinyl as an acceptor to tetraphenylethene in forming AIE nanodots with large two-photon absorption cross section, which can be applied as nano-photosensitizers for two-photon bioimaging and photodynamic therapy with high cellular uptake. In a separate study, Hu et al. [84] incorporated the [PhC=C(CN)_2_] moiety as an electron acceptor to the alkoxyl- tetraphenylethene (as electron donor) in obtaining red emissive AIE photosensitizer, which was then used to form AIE nanodots by Alifu et al. [87].

It is found that the formulation of AIE photosensitizer into nanodots can further improve the performance of AIE photosensitizer for PDT. In the nanodot formulation, AIE photosensitizer molecules are well aggregated inside nanoparticle and therefore restricting the intramolecular motions/vibrations, which resulted in the enhanced intersystem crossing, leading to the increased of SOG efficiency with improved PDT performance. For example, Liu et al. [88] introduced the quinolinium iodide to a TPE molecule to make a mitochondria-specific AIE photosensitizer (tetraphenylethene-Qu^+^), thanks to formation of *D*-*π*-*A* structure and positive surface charge of quinoline (Qu^+^) molecules. The as-designed photosensitizer molecules were further encapsulated into the bovine serum albumin capped calcium carbonate nanoparticles (i.e., 100 nm) through mineralization process. The as-synthesized nanoparticles showed pH-responsive behavior due to decomposition of CaCo_3_ nanoparticles by lysosome inside the cancer cells (Fig. 4b). Zhao’s group [89] also have designed a new AIE photosensitizer called TPMD by *D*-*A* structure approach, followed by formulation of the photosensitizer into nanodots using Pluronic F127 for multimodal PDT/photothermal therapy (PTT) of cancer cells (Fig. 4c). Ma et al. [90] also have utilized the same *D*-*π*-*A* structure to design new AIE photosensitizer, called DSABBT, by combining 4-(diethylamino)-salicylaldehyde and 4,7-bis(4-aminophenyl)-2,1,3-benzothiadiazole. DSABBT molecules were further encapsulated into amphiphilic DSPE-PEG2000-cRGD to form the 105 nm nanoparticles for image-guided PDT. The D-A structure could also be used to design two-photon AIE photosensitizer, as illustrated by Tang’s group [91]. The proposed AIE photosensitizer was encapsulated into nanoparticles using 1,2-dioleoyl-sn-glycero-3-phosphoethanolamine and 1,2-dioleoyl-3-trimethylammonium-propane. The AIE-PS nanoparticles showed simultaneous generation of singlet oxygen and hydroxyl radicals through different pathways, which is beneficial for effective PDT (Fig. 4d). More recently, we have successfully developed the gold nanostars-AIE theranostic nanodots to further enhance the fluorescence brightness and SOG efficiency toward image guided PDT [21].

The hypoxia is a state that the concentration of oxygen in the tissue is overly low. Since PDT is an oxygen-dependent process, the concentration of O_2_ inside the cells decreases during the process of PDT, leading to the reduced efficiency of SOG generation. Additionally, hypoxia often results in aggregation of conventional photosensitizers (especially those with aggregation-caused quenching properties), further suppressing the efficacy of PDT treatment. To address this challenge, various strategies including regulating the tumor microenvironment and using non-reactive oxygen carriers (e.g., hemoglobin) have been developed [92]. On the other hand, Wan et al. [93] reported that the introduction of anion-*π*^+^ moiety to the backbone of AIE molecules could result in the formation of AIE photosensitizers with considerable singlet oxygen generation under hypoxia condition.

**Fig. 5:**
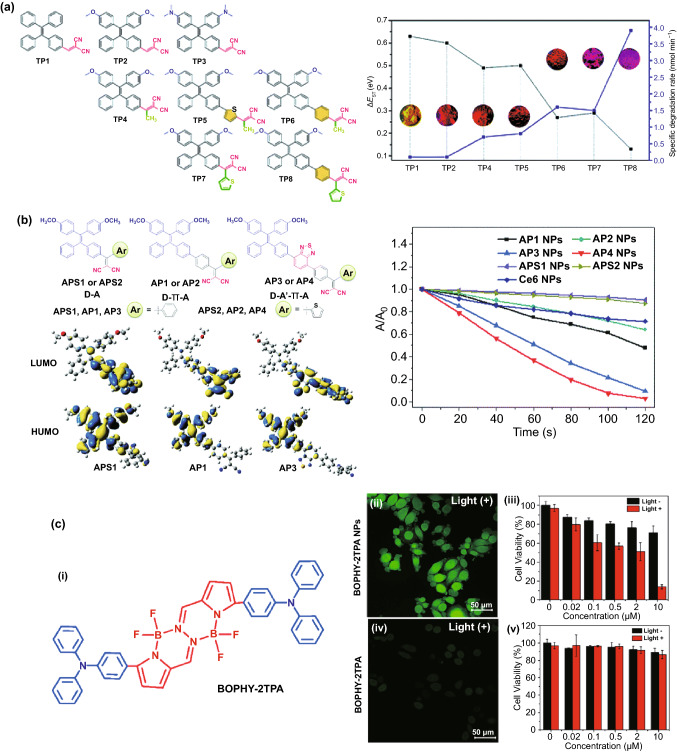
**a **Left: Chemical structure of different Type II AIE photosensitizers, Right: The Δ*E*_ST_ values (black curve), specific degradation rates (degradation rate per absorption area of the PS, 400–700 nm) and solid power images under 365 UV light illumination (inset), reproduced from Ref. [95] with permission from Royal Society of Chemistry. **b** Left: Chemical structures and HOMO–LUMO distributions, and Right: relative degradation of 9,10-anthracenediyl-bis(methylene) dimalonic acid (ABDA) in the presence of different photosensitizers, reproduced from Ref. [97] with permission from Royal Society of Chemistry. **c** (i) Chemical structure of BOPHY-2TPA AIE photosensitizer molecule, (ii) fluorescence image of intracellular ROS generation in HeLa cells detected by DCFH-DA as singlet oxygen probe, and HeLa cell viability by (ii, and iii) BOPHY-2TPA nanoparticles and (iv, v) BOPHY-2TPA molecule, reproduced from Ref [99]. with permission of American Chemical Society. (Colour figure online)

In addition, the distances between the introduced donor/acceptor groups as well as the torsional angle and the electron cloud distribution can be controlled to further improve the performance of AIE molecules for SOG. For example, it has been shown that shorter distance could be more favorable for charge transfer in the excited molecule which results in smaller ΔE_ST_. Therefore, the longer distance between donor and acceptor could be more favorable for charge transfer in the excited molecule which results in smaller Δ*E*_ST_ [94]. Xu et al. [95] have designed different types of AIE photosensitizers to tune the distance between donor and acceptor by introducing a linker (Fig. 5a). It has been shown that by introducing different donor/acceptor groups to TPE core, Δ*E*_ST_ could be well tuned and singlet oxygen generation could be altered due to increase in separation of HOMO–LUMO. Zhao et al. [96] have used this approach to design an AIE photosensitizer with two long alkyl chains and two positively charged amine groups that could well interact with bacteria membrane. It has been used for bacterial imaging and killing without washing step owing to the AIE properties and high ROS generation ability. An increase in the torsional angle could lead to the better separation of HOMO–LUMO and consequently, decrease in Δ*E*_ST_ [11, 95]. Additionally, the SOG of AIE photosensitizer can be enhanced by introducing an auxiliary acceptor. Wu et al. [97] (Fig. 5b) have improved the photosensitizing properties of AIE by introducing benzothiadiazole as an auxiliary group to form a *D*–*A*′–*π*–*A* structure, while methoxy-substituted tetraphenylethylene-phenyl-dicyanovinyl is used as an electron donor, *π* spacer, and an electron acceptor, respectively. This approach facilitates the separation of the HOMO–LUMO that has a direct effect on ISC and SOG properties of the AIE molecule.

The second approach is to introduce the AIE-moiety to a non-AIE molecules (e.g., porphyrin) and convert it to an AIE-based photosensitizer. For example, Rananaware et al. [98] have decorated porphyrin with four tetraphenylethene molecules. The as-obtained nanostructures showed aggregation-induced emission properties as well as singlet oxygen generation ability. In another work, bis(difluoroboron)-1,2-bis ((1H-pyrrol-2-yl)methylene) hydrazine (BOPHY), which is a highly emissive ACQ fluorophore, was conjugated to two units of triphenylamine to form the AIE photosensitizer based on donor–acceptor–donor (*D*–*A*–*D*) Structure [99]. The as-designed AIE photosensitizer was encapsulated with biocompatible Pluronic P123 into water soluble nanodots for PDT treatment of HeLa cells. The results revealed that the as-formulated AIE nano-photosensitizer dramatically enhanced the internalization of nanodots by the HeLa cells, leading to higher rate of intracellular SOG generation (Fig. 5c). Although this approach seems very interesting and promising due to existence of many ACQ photosensitizers, finding the right AIE moiety to be paired with the ACQ photosensitizers as well as keeping its photosensitization ability upon chemical modification seems to be a challenging step, restricting further exploration of new AIE photosensitizers via this approach.

#### Metal Nanoclusters-Based Photosensitizers

**Fig. 6:**
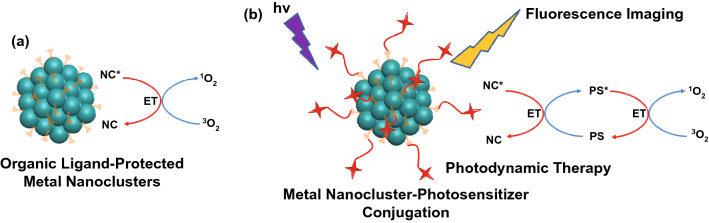
**a** Organic ligand-protected metal nanocluster for singlet oxygen generation. **b** Metal nanocluster-photosensitizer conjugation as multifunctional nanomaterial for simultaneous florescence imaging and photodynamic therapy. Nanocluster acts as donor and PS acts as acceptor in the energy transfer (ET) process

In recent years, ultrasmall metal nanoclusters (< 2 nm in core) such as gold and silver NCs have attracted extensive research interests both in basic and applied science owing to their unique physiochemical properties such, well defined composition (monodispersity with atomically precise formulas) and molecular-like absorption and emission [100, 101]. The synthesis strategies of gold and silver nanoclusters have been well documented in several recently published review articles [69, 102–107]. In particular, the strategies of using biomolecules (e.g., DNA and protein) as templates [67, 68, 71, 108, 109] to form the multifunctional metal nanoclusters is of outmost interest due to their suitability for a range of biomedical applications from sensing [70, 110, 111], to bioimaging [112–114], and drug/gene delivery [115–117]. However, the singlet oxygen generation property of metal nanoclusters and its practical applications for PDT, has been less well reported [12, 65, 118]. This section summarizes recent progress of developing metal nanoclusters-based photosensitizers and their applications in PDT. Analogous to a conventional photosensitizer, metal nanoclusters possess both photoluminescence property and ROS generation ability. In general, there are two ways to exploit the metal nanoclusters as theranostic agents for simultaneous bioimaging and PDT. The first one is employing the intrinsic photoluminescence and ROS generation properties of metal nanoclusters for image-guided PDT, while the second approach is to conjugate the metal nanoclusters as imaging probes with another photosensitizer to form the multifunctional theranostic agents (Fig. 6). As compared to the conventional photosensitizers, metal nanoclusters are featured with superior ROS generation efficiency, good aqueous solubility, and excellent photostability, thus exhibiting great potential as a new generation of nano-photosensitizer for PDT applications. Although promising progress has been made for the synthesis of photoluminescent Au nanocluster, it should be mentioned that the blue and red emitting Au nanoclusters might not be practical as a fluorescence imaging agent for clinical applications because they have relatively low tissue penetration ability in the short excitation and emission wavelength.

**Fig. 7:**
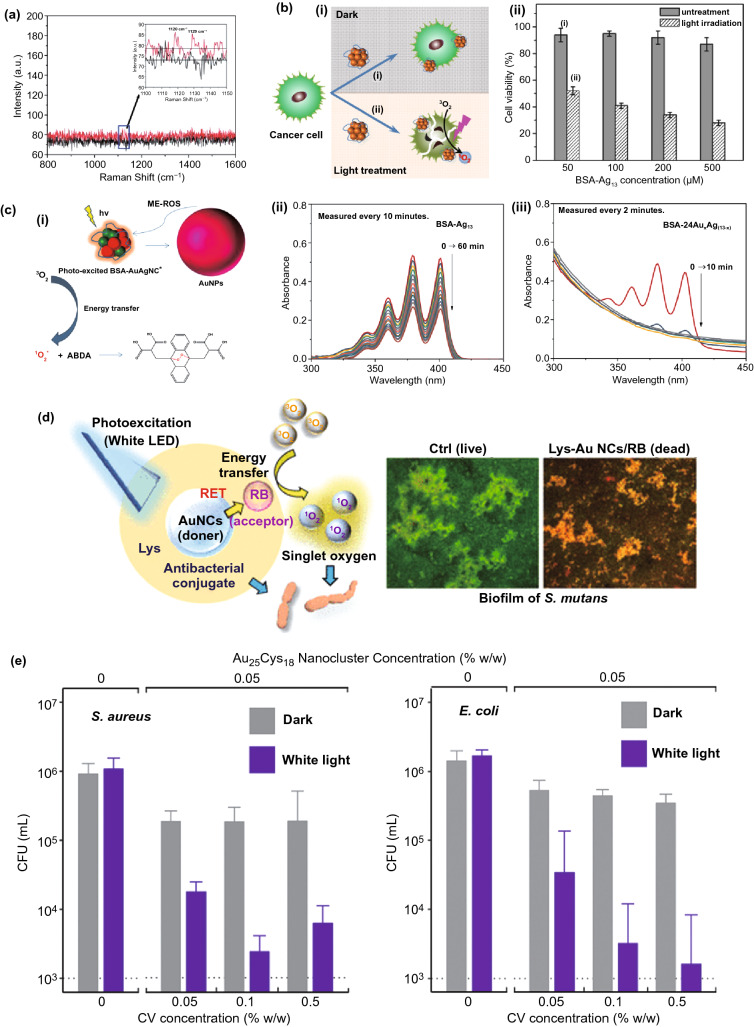
**a** Raman spectral signals for oxygen molecules adsorbed on blue emitting BSA-capped Au nanoclusters, reproduced from Ref [119]. with permission from Royal Society of Chemistry. **b** The cytotoxicity effects of BSA-capped Ag_13_ nanoclusters on MCF-7 cancer cells as a biocompatible PDT agent for cancer treatment, reproduced from Ref [12]. with permission from Wiley Online Library. **c** (i) Mechanism of metal-enhanced ROS in the AuAg nanoclusters synthesized by galvanic replacement approach, and Degradation of ABDA solution (50 × 10^−6^ M) in the presence of 200 μM of (ii) BSA-Ag13NC, and (iii) BSA-AuAg alloy nanocluster, reproduced from Ref [65]. with permission from Elsevier. **d** Lysozyme capped Au nanocluster-Rose Bengal conjugate as an effective photosensitizer to inhibit the formation of *Streptococcus mutans* biofilm, reproduced from Ref [126]. with permission from American Chemical Society. **e** Concentration effect of AuNC-Crystal violet conjugate incorporated in the ultra-high molecular weight polyethylene on its antibacterial properties against (i) *Staphylococcus aureu*s and (ii) *Escherichia coli* under visible light (375 lx), reproduced from Ref. [127] with permission from Royal Society of Chemistry. CV: Crystal Violet

The feasibility of gold nanoclusters interacting with molecular oxygen to produce singlet oxygen was first explored in 2012 [119]. By using bovine serum albumin as the template, Das et al. synthesized blue and red emitting gold nanoclusters, which showed an opposite change in fluorescence intensity in the presence of molecular oxygen. The generation of singlet oxygen accompanied by enhancement of fluorescence intensity of the blue emitting Au nanoclusters was verified by using diaminobenzidine as an indicator. Raman spectra of the oxygen saturated blue emitting Au nanocluster solution was also measured, but the signal of singlet oxygen was not obvious (Fig. 7a). Kawasaki et al. found that atomically precise Au_25_(SR)_18_ cluster (there are 25 gold atoms and 18 thiolates in a single cluster) was able to generate singlet oxygen efficiently. The characteristic emission of singlet oxygen was clearly observed at − 1276 nm by fluorescence spectroscopy. Three singlet oxygen selective probes (i.e., DAB, 1,3-diphenylisobenzofuran, and 9,10-dimethylanthracene) were also used to confirm the production of singlet oxygen. In vitro studies with Hela cells demonstrated the feasibility of Au_25_(SR)_18_ cluster for NIR induced PDT [120]. In another separate study, Au_25_(SR)_18_ was attached to metal–organic frameworks (MOFs) of Fe_3_O_4_/ZIF-8 nanoparticles due to its NIR induced single oxygen generation ability. The nanocomposite material is unique due to its imaging-guided enhanced synergistic therapeutic effect [121]. The ligand effect of metal nanocluster on SOG has also been studied recently. Yamamoto et al. synthesized Au_25_ cluster protected by BSA and glutathione, respectively, and studied their SOG property using a fluorescent ^1^O_2_ probe methotrexate. It has been found that BSA-templated Au_25_ generates ^1^O_2_ about 6 times faster than its GSH-protected analogue [122]. The SOG property is not restricted to Au nanoclusters only. A recent study by Tan’s group has shown that BSA-Ag_13_ nanocluster containing 13 Ag atoms in a single cluster possess excellent ^1^O_2_ generation capability as compared to previously developed nanocluster-based photosensitizer [12]. Using ABDA as the indicator and compared to commercial photosensitizer Rose Bengal, the BSA-Ag_13_ nanocluster reports a ^1^O_2_ quantum yield of 1.27. Due to the protection of the biogenic template, the BSA-Ag_13_ nanocluster possesses low dark cytotoxicity and has been demonstrated for cancer therapy using MCF-7 cancer cells as a model (Fig. 7b) [12]. Most recently, the same group has developed the protein-protected gold/silver alloy nanoclusters through galvanic replacement reaction and unravels their correlation with photoluminescence. It was found that plasmonic gold nanoparticles (> 10 nm) were also formed simultaneously by photobleaching of the BSA-AuAg nanoclusters, leading to significant metal enhancement effect to the ^1^O_2_ generation rate (Fig. 7c) [65]. To further improve the therapeutic effect of metal nanoclusters, a nucleus targeting Au nanoclusters was also reported in by using TAT peptide as the protecting agent for Au nanoclusters. Confocal laser scanning microscopy results show that a significant fraction of the as-synthesized Au nanoclusters enter the nucleus. The TAT peptide templated Au nanoclusters also worked as DNA delivery cargoes showing 90% cellular uptake and 80% gene transfection efficiencies in HeLa cells [123].

Other than the intrinsic ROS generation property, metal nanoclusters also contribute to photodynamic therapy in other different ways. Due to its strong photoluminescence, Au nanoclusters have been utilized to combine with conventional photosensitizers for fluorescence imaging assisted PDT. For example, BSA-templated Au nanocluster coated with SiO_2_ was further conjugated with a conventional photosensitizer Ce6 to constitute a multifunctional theranostic agent. The silica-coated Au nanocluster achieved higher loading efficiency of Ce6 and resulted in no non-specific release of Ce6 during circulation and enhanced cellular uptake efficiency [124]. Similar strategy has also been adopted by grafting folic acid conjugated PEG (polyethylene glycol) on the surface of glutathione protected Au nanocluster where folic acid provides tumor-targeting property, PEG works for photosensitizer Ce6 loading and Au nanocluster serves as an imaging probe. In vitro studies showed enhanced cellular uptake of the composite material and good PDT effectiveness toward MGC-803 cells while in vivo studies with MGC-803 tumor-bearing nude mice showed the composite material with superior penetration and retention behavior in tumors and preserved features of renal clearance and stealthy to reticulo-endothelial system. In another study, NIR-emitting Au nanoclusters was synthesized with lipoic acids which renders the as-synthesized Au nanocluster with the tumor-targeting property. The lipoic acid protected Au nanoclusters was further linked with a photosensitizer protoporphyrin IX via (*N*-ethyl-*N*′-(3-(dimethylamino)propyl)carbodiimide/N-hydroxysuccinimide) (EDC/NHS) chemistry for selective PDT. Interestingly, this composite PDT agent showed 80% triplet quantum yield, which was a great improvement as compared to implementing the photosensitizer alone (63%). The efficacy of the composite PDT agent was further confirmed by in vivo tumor imaging and histopathology study [125]. Recently, Okamoto et al. [126] have synthesized a lysozyme Au nanocluster-Rose Bengal conjugate, to make a biocompatible and efficient photosensitizer under visible light. Au nanocluster plays as a donor and Rose Bengal acts at acceptor in this conjugate, leading to high rate of singlet oxygen generation, and in turn, decreasing the *Streptococcus mutans* biofilm formation under white LED light irradiation (Fig. 7d). The application of metal nanocluster-photosensitizer conjugate is not limited to the solution. Wu et al. [127] have reported the incorporation of Au nanoclusters and crystal violet into the ultra-high molecular weight polyethylene to make antibacterial film. The as-designed film showed a great antibacterial effect against *Staphylococcus aureus* and *Escherichia coli* (Fig. 7e), thanks to high energy transfer rate from Au nanoclusters to crystal violet molecules, and in turn, high SOG under white light illumination.

In summary, ultrasmall metal nanoclusters have demonstrated its potential for photodynamic therapy with the excellent singlet oxygen generation capability and unique photoluminescence property both in vitro and in vivo. However, there are still much work yet to be done for this new type of nano-photosensitizer to be implemented for clinical applications, such as tailoring the singlet oxygen generation property from the design point of view (e.g., response to desired wavelengths and with high ^1^O_2_ efficiency), a universal benchmark for PDT performance evaluation, as well as more in-depth understanding of the interaction between metal nanocluster and the biological systems.

#### Carbon Dots-Based Photosensitizers

**Fig. 8:**
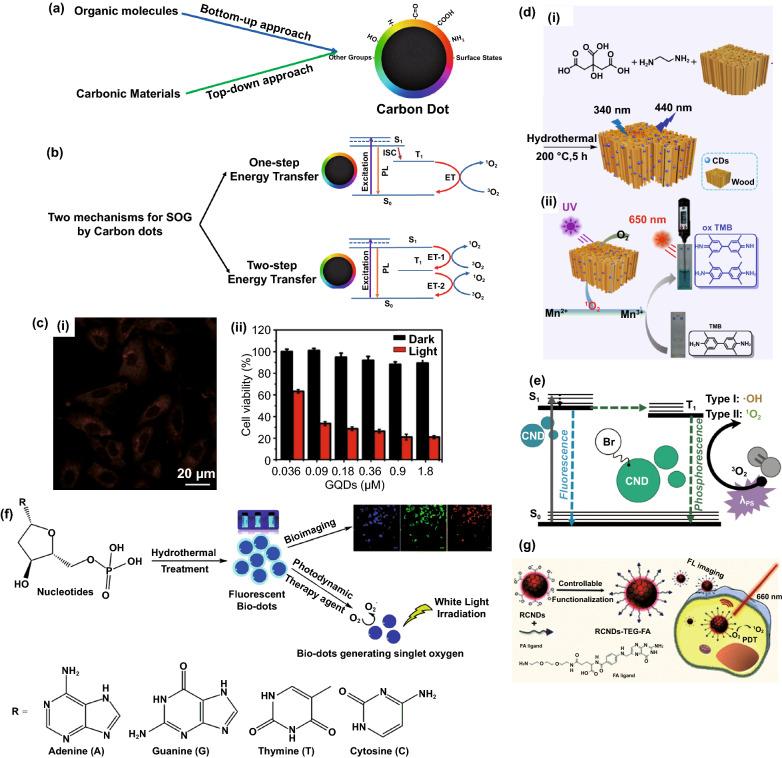
**a** Synthesis approaches of carbon dots having different functional groups on their surface. **b** Different mechanisms for singlet oxygen generation by carbon dots: One-step energy transfer like common photosensitizer and two-step energy transfer mechanism proposed by Ge et al. (adapted and reproduced from Ref. [147] with permission from Springer-Nature). **c** Fluorescence imaging of HeLA cells and corresponding cytotoxicity effects of the graphene dots on HeLa cells, reproduced from Ref. [147] with permission from Springer-Nature, **d** (i) Synthesis of carbon dots confined in the wood structure, and (ii) Schematic Illustration of photothermal signal-amplified detection of Mn^2+^ based on the detection system of CDs@wood and 3,3,5,5-tetramethylbenzidine, reproduced from Ref [153]. with permission from American Chemical Society. **e** Jablonski diagram for brominated carbon dots with ROS generation ability, reproduced from Ref. [154] with permission from Royal Society of Chemistry. **f** Schematic illustration of amino acids-derived biodots for fluorescence imaging and photodynamic therapy under irradiation of visible light, reproduced from Ref. [134] with permission from Royal Society of Chemistry. **g** Folic acid-functionalized carbon dots synthesized from polythiophene phenylpropionic acid for fluorescence imaging and PDT under 660 nm laser, reproduced from Ref. [158] with permission from Royal Society of Chemistry

Carbon-based nanomaterials including graphene-family, carbon nanotubes, carbon black and carbon dots have received many attentions due to their unique physical, chemical and mechanical properties, which make them suitable for biomedical applications. Among them, carbon dots are of particular interest owing to their high biocompatibility, water solubility and low dark toxicity, multi-excitation fluorescence as well as easy surface modification due to the presence of various functional groups on their surfaces [128, 129]. The first report on the discovery of carbon dots was dated back to 2004 during isolation and purification of carbon nanotube [130]. Carbon dots, which typically are quasi-spherical and have an average size less than 10 nm, could be synthesized via two main approaches: top-down approach and bottom-up approach (Fig. 8a). In a top-down approach, carbon dots are produced by laser ablation, discharge, and electrochemical oxidation, which involves the use of sophisticated high-tech equipment and are thus more expensive. In a bottom-up approach, carbon dots are synthesized by more cost-effective methods such as solvothermal or hydrothermal carbonization, microwave pyrolysis, and acid hydrolysis [131]. Recently, natural resources including biomass waste like fruit peels, biomolecules such as DNA and amino acids, have been utilized to form multifunctional carbon dots with tailored biofunctionalities (also known as biodots) [132]. These biodots are especially suitable for biomedical applications due to their intrinsic physiochemical properties and integrated biofunctionalities such as antimicrobial, antitumor, etc. [133–139]. Furthermore, the fluorescence spectra of carbon dots /biodots could be tuned according to the precursor composition (e.g., amino acids combination) [140], and some may exhibit multi-excitation properties [141–143] leading to a wide range of applications.

As CDs have excellent light absorption and photoluminescence as well as efficient charge transfer from/to nearby materials and species [144–146], it is expected that some CDs would be able to generate reactive oxygen species (ROS) via energy transfer from their excited triplet states to surrounding oxygen molecules (Fig. 8b). However, the ROS generation ability of carbon dots cannot be predicted from the nature of the organic precursor. In one of the earliest works, Ge et al. [147] used the polythiophene quaternary ammonium as precursor to synthesize carbon dots via a hydrothermal method with a calculated SOG efficiency of 1.3 (a relative value obtained by comparing it to the reference material, i.e., Rose Bengal with a SOG efficiency of 0.75), a value which is much higher than most of the conventional organic photosensitizers. These carbon dots absorbed broadly in the visible range and showed strong emission at 680 nm and have been applied for simultaneous imaging and PDT both for in vitro and in vivo (Fig. 8c). In a follow-up work, different polythiophenic precursors were used to synthesize carbon dots and their SOG efficiencies were compared. The carbon dots synthesized with polythiophene benzoic acid precursor emitted in the red and NIR regime, exhibiting a SOG efficiency of 0.27. More interestingly, a high photothermal conversion efficiency (36.2%) was also observed. This carbon dots was thus used for combined imaging/PDT/PTT [148]. However, CDs generally do not absorb the NIR region for efficient and practical PDT as light of longer wavelengths is preferred due to deeper tissue penetration [149]. To address this issue, Jia et al. [150] applied a self-assembly strategy to synthesize carbon dots nanospheres with negatively charged amphipathic sodium dodecyl benzene sulfonate (SDBS) as a linker. The resulted nanospheres exhibited SOG efficiency of 0.45 under excitation of 671 nm laser and have been applied for PDT of 4T1 cells. In another work [151], the same group synthesized carbon dots with magnetofluorescence properties using manganese(II) phthalocyanine, which is a photosensitizer molecule itself as the precursor. The as-obtained self-assembled carbon dots (with SOG efficiency of 0.4) using 1, 2-Distearoyl-sn-glycero-3-phosphoethanolamine-Poly(ethylene glycol) exhibited both NIR fluorescence (maximum peak at 745 nm) and T1-weighted magnetic resonance (relativity value of 6.97 mM^−1^ s^−1^) which could be used for simultaneous fluorescence imaging and MRI. Interestingly, the carbon dots also possess catalase property that can convert hydrogen peroxide (H_2_O_2_) to O_2_. Feng et al. [152] also synthesized a porphyrin-like structure carbon dots by a simple cyclization reaction of 4-formylbenzoic acid and pyrrole and cross-linking with p-phenylenediamine at room temperature. The as-synthesized carbon dots possessed high antibacterial properties under 638 nm laser irradiation. In an interesting work, Su et al. [153] have synthesized carbon dots confined into wood (CDs@wood) using in-situ solvothermal synthesis approach starting from ethylenediamine and citric acid as precursors. This approach prevents the aggregation-induced quenching of carbon dots, resulting in 3-fold higher singlet oxygen generation as compared to that carbon dots without confinement in wood under 660 nm laser irradiation. The as-designed nano-photosensitizer was used to detect Mn^2+^ ions through a combined photodynamic/photothermal approach, where the LOD of 44.6 nM was found for the method described in Fig. 8d. On the other hand, Geddes et al. [154] reported the synthesis of brominated carbon dots with photosensitization through either electron transfer (type I) or energy transfer (type II) pathways, to generate both singlet oxygen and hydroxyl radicals under irradiation. While the non-brominated carbon dots in this study (without boron) possess only fluorescence property, the presence of boron in their structure has been shown to facilitate the ISC and therefore the ability of ROS generation (Fig. 8e).

The precursors that have been used for the synthesis of CDs with the ability of SOG are not limited to polythiophene derivatives. Yao et al. [155] have used the mixture of citric acid and formamide as precursors to form the CD-based photosensitizer that showed SOG property under 532 nm light. Carbon powders have also been used to prepare CDs that showed good PDT effect for human prostate adenocarcinoma (Du145 and PC3) cell cultures in vitro under UV light irradiation [156]. There are also a few attempts to synthesize CDs with intrinsic ability of SOG using photosensitizer in combination of different molecule as precursors. For example, Li et al. [157] have synthesized CDs with intrinsic PDT effect starting from mono-hydroxylphenyl triphenylporphyrin and chitosan (sugar), which showed acceptable SOG property as well as good water solubility and photostability. More recently, our group has conducted systematic study on the use of natural nucleotides as precursors for the synthesis of theranostic biodots with intrinsic fluorescence and singlet oxygen generation for bioimaging and photodynamic therapy [134]. The as-synthesized deoxyadenosine monophosphate biodots not only show a remarkable singlet oxygen quantum yield of 1.20 surpassing that of the conventional photosensitizer Rose Bengal (0.75), but also display high fluorescence quantum yield (12.4%) and good photo-stability (91.9%), which are the important attributes for theranostic PDT agents (Fig. 8f). In another work, Ji et al. [158] have synthesized folic acid-functionalized carbon dots using polythiophene phenylpropionic acid as precursor, where the singlet oxygen quantum yield of 0.4 was observed. The as-synthesized functionalized CDs showed a great cellular uptake due to presence of folic acid on its surface (Fig. 8g).

## Boosting the Singlet Oxygen Generation Rate by Plasmonic Nanostructures

Up to now, we have covered three types of newly developed nano-photosensitizers with unique physiochemical properties that could overcome the limitations of classical photosensitizers. As mentioned earlier, other than exploring the design of new photosensitizers, another feasible method is to employ the plasmonic (e.g., gold/silver) nanostructures to enhance SOG of photosensitizer based on a physical phenomenon called metal-enhanced singlet oxygen generation (ME-SOG). In this session, we will discuss the general mechanism of ME-SOG, highlight different types of metal-enhanced photosensitizer systems and identify the critical parameters in designing the metal enhancement systems beyond singlet oxygen generation (e.g., fluorescent enhancement and NIR excitation).

### Mechanism of Metal-Enhanced Singlet Oxygen Generation

**Fig. 9:**
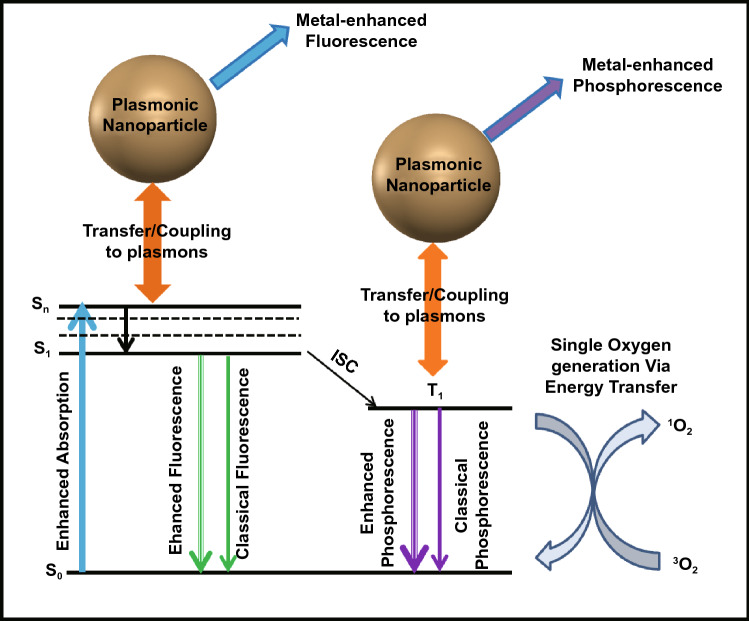
Jablonski Diagram for a photosensitizer molecule in the vicinity of a metal nanostructure. *S*_0_, *S*_1_, and *S*_*n*_ are referred to the different levels of singlet state. *T*_1_ is the lowest level of triplet state, and ICS stands for intersystem crossing process

Since the first observation of ME-SOG by Geddes et al. [18], many attempts have been made to study the ME-SOG and investigate the possible mechanisms to explain this interesting phenomenon, which is opposite to the common belief about the quenching effects of metal nanoparticles for both fluorescence and photosensitization [159–161]. Although the exact mechanism for ME-SOG as well as the metal-enhanced fluorescence (MEF) have not been concluded yet, one possible origin is the increase in the rate of excitation as a result of the enhanced electric field around the metal nanostructures (Fig. 9). Consequently, the rate of ISC could be enhanced and thus results in enhanced energy transfer from triplet-excited photosensitizer (PS) molecules to the surrounding oxygen and/or water molecules to produce singlet oxygen and other ROS. ME-SOG have some similarity with MEF as both phenomena are a consequence of the near-field interaction between the plasmonic nanostructures and photosensitizer (or fluorophore) molecules. However, ROS generation is a non-radiative process while MEF relies on radiative processes. Hence, the effective factors on these two processes could be similar while their contributions in each process might be different. For example, the fluorescence of the fluorophore quenched in a very short distance between the metal nanostructures and the fluorophore molecules. However, the ROS generation might not be quenched, necessarily. Figure 9 shows the simple Jablonski diagram for a photosensitizer molecule in the vicinity of a metal nanostructure, where different radiative and non-radiative pathways are affected by the plasmonic influence of a metal nanoparticle.

The size and shape of metal NP is pivotal in affecting the performance of ME-SOG. Different structures of metal nanoparticles have been constructed to fully exploit the potential of ME-SOG, which can be mainly categorized into two metal enhancement systems, i.e., the planar and colloidal systems with photosensitizer. Despite a lot of efforts on developing planar metal-PS hybrid systems with ME-SOG effect, their applications in biomedical field would be limited to the PDT of skin-related diseases [162, 163] and antibacterial films [164]. Hence, developing colloidal metal NP-photosensitizer hybrid systems with ME-SOG effect is essential for efficient PDT of different cancers. In this section, the theory of ME-SOG, different metal nanostructures used to enhance the ROS generation of photosensitizer, as well as the effective parameters of each system will be discussed.

### Metal-Enhanced Singlet Oxygen Generation in Planar Systems

**Fig. 10:**
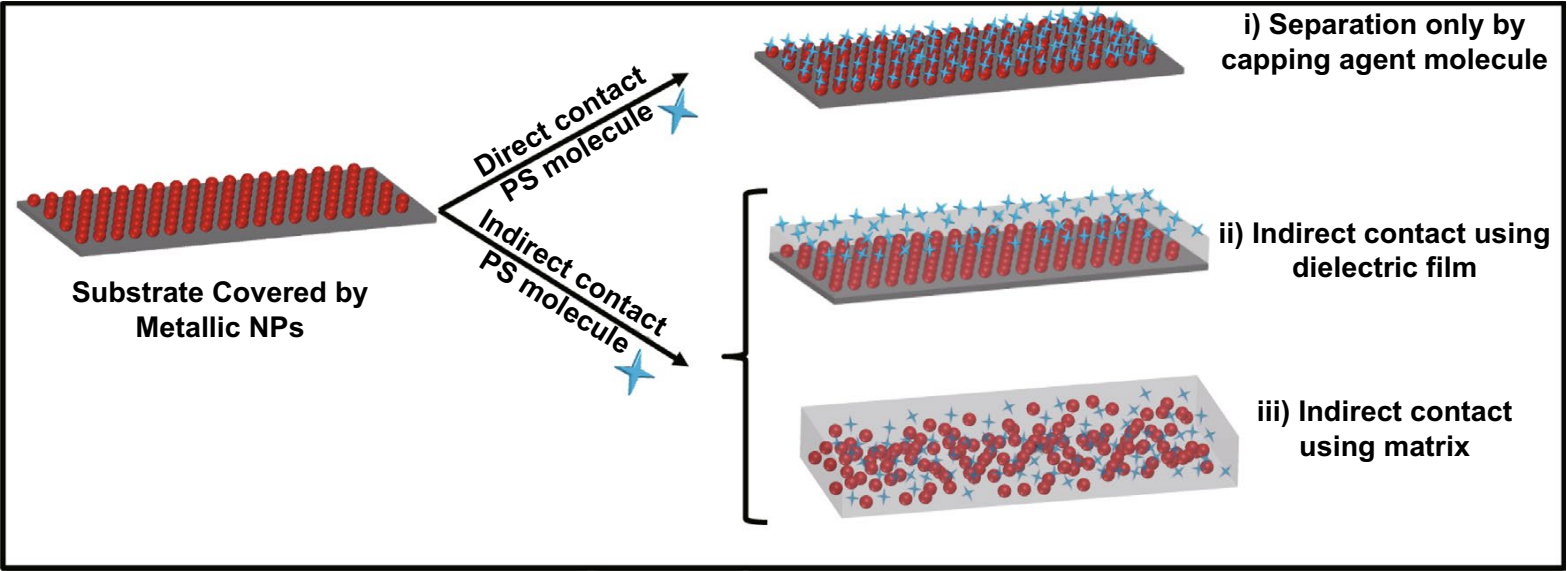
Different approaches for fabrication of planar system with metal-enhanced singlet oxygen: (i) Direct contact between photosensitizer molecules and metal nanoparticles, (ii) Using dielectric (e.g., silica or polymer) film as spacer between the metal nanoparticles and photosensitizer molecules, and (iii) Using a dielectric matrix (e.g., polydimethylsiloxane) containing both metal nanoparticles and photosensitizer molecules

Planar metal-photosensitizer system is the first approach that was used for ME-SOG by Geddes et al. in 2007 [18]. The planar plasmonic nanostructures for ME-SOG are typically manufactured by the creation of metal islands on a silicon or glass substrate. The metal islands could be synthesized by different approaches like electrodeposition [165] where the size of the final island can be controlled by deposition time and lithography technique [166] which enables the fabrication of metal nanostructures with different size and shapes. As the distance between the metal nanostructures and photosensitizer can be flexibly controlled, therefore planar systems are more useful for fundamental studies, for instance, to study the distance-dependent behavior of ME-SOG. ME-SOG in planar metal-photosensitizer system have been studied via different approaches, which is schematically shown in Fig. 10. Generally, there are two main approaches for developing planar systems: direct contact and indirect contact. In first approach, PS molecule bind directly to the surface of pre-deposited metal nanoparticles, while in indirect contact approach, the distance between the photosensitizer molecule and pre-deposited metal nanoparticle is controlled by using dielectric layer or a dielectric matrix.

In the early reports on the observation of metal-enhanced ROS on the planar system, ME-SOG was studied by fabricating the silver islands on glass substrate with a sandwich structure, containing the Rose Bengal as photosensitizer molecule and sensor green reagent as the singlet oxygen indicator. It was found that the rate of SOG could be enhanced 3-fold in the presence of silver island film as compared to the control sample, which is the glass substrate with no SIF. Further studies indicated that the absorbance of Rose Bengal was enhanced in the vicinity of SIF, providing direct evidence for the enhanced excitation rate as a consequence of the enhanced electric field around the silver island film [18]. In another study, the effect of intrinsic singlet oxygen generation quantum yield (SOG-QY) on the free space (headroom) of ME-SOG was investigated. It was found that the SOG-enhancement factor has an inverse relationship with the SOG-QY where SOG rate for quinidine (SOG-QY = 0.08) was enhanced about 26.6 times while the enhancement factor for acridine (SOG-QY = 1) was only 1.83-fold [17].

Due to the similarity between the MEF and ME-SOG, the spectral overlap between the metal substrate and the PS molecule was identified as an important factor on the final ROS enhancement factor. Karollin and Geddes [167] have reported that both singlet oxygen and superoxide anion radical generation could be enhanced using the same approach by locating Rose Bengal and acridine in the vicinity of silver island film. However, the enhancement factor of SOG by Rose Bengal was higher than the enhancement factor of superoxide anion radical, which could due presumably to a lesser spectral overlap between the absorbance of the acridine and extinction spectra of the silver island film. Another effective factor is the power of illuminated light (or laser). The ROS enhancement factor changes nonlinearly with the power of illuminating light, which trend is similar to that in the MEF. Furthermore, the numerical simulation of the near-field electric field around silver nanoparticles has verified that the volume of the enhanced electric field around plasmonic nanoparticles increase exponentially with increasing power of the illuminated light, that is, so-called excitation volumetric effect [167, 168].

The singlet oxygen generation rate can be measured via two main approaches, indirect measurements using selective probes (e.g., ABDA molecule) or direct measurement of phosphorescence that comes from the as-produced singlet oxygen (i.e., at 1270 nm wavelength). Ragas et al. [19] has studied the metal-enhanced phosphorescence of singlet oxygen (at 1270 nm wavelength) generated by C_60_ in the presence of silver island film where the maximum enhancement factor of 35 was observed for 2 min silver island film deposition time. The deposition time of silver islands is an effective factor in the planar system as the increase in deposition time can alter the size of silver islands as well as the distance between the islands. These two parameters (i.e., size and distance) are important in the planar ME-SOG systems in affecting the enhanced electric field around the silver island film. However, the measurement of phosphorescence of singlet oxygen at 1270 nm is not an indicative for the actual enhancement of the SOG rate since it contains some false-positive results. This is because the presence of metal nanostructure not only affects the intersystem crossing rate and thus the rate of SOG, but also enhances the radiative decay rate due to the metal-enhanced phosphorescence (MEP) effect [169–171]. Hence, the singlet oxygen phosphorescence enhancement factor is not the same as the enhancement factor of SOG. The actual SOG enhancement factor in a ME-SOG system can be less than the observed singlet oxygen phosphorescence enhancement factor in the same system.

Similar to MEF system, it is expected that the ME-SOG would have a distance-dependent behavior. Firstly, this is due to the nonlinear trend in the propagation of electric field in the surrounding environment, where the maximum electric field is observed at the surface of the metal nanostructure, and it decreases exponentially with the increasing distance from the surface. Secondly, the nonlinear non-radiative energy transfers from the excited PS molecules to the metal surface, which has an inverse trend with the separation distance between the PS molecule and metal surface. To investigate the effect of distance on ME-SOG systematically, dielectric layer is normally introduced to the surface of metal nanoparticles in the planar system. Till date, different approaches have been used to control the distance between the PS molecules and the surface of the metal substrate in nm-scale [172]. The most common method is to use the silica as spacer which provides optical transparency, easy preparation, and stability while requires delicate control of thickness in nm-scale through cautious optimization and instrumental characterizations [173–175]. The other method is through the self-assembling of polyelectrolytes with different charges using the layer-by-layer (LBL) deposition process. This approach provides the controlled thickness in the nanometer range as well as to incorporate the environmental stimuli-responsivity functions onto the film depending on the type of selected polyelectrolytes used to bind the charged PS molecules [176–180].

**Fig. 11:**
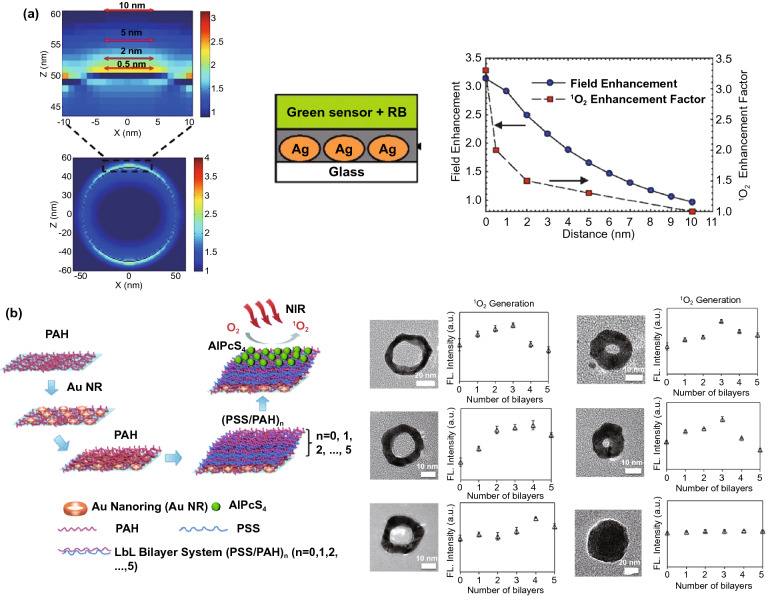
**a** Distance-dependent of ME-SOG for Rose Bengal on silver island film coated with silica layer as a spacer, reproduced from Ref. [17] with permission from National Academy of Sciences. **b** Distance-dependent of ME-SOG for AlPcS_4_ on glass substrate containing Au nanoring coated with different PAH/PSS bilayers, reproduced from Ref. [181] with permission from Royal Society of Chemistry

The distance-dependent behavior of ME-SOG in the planar system was first studied by Zhang and co-workers [58] (Fig. 11a). Unlike the MEF system where the fluorophore quenches at a very short distance (< 2 nm) from the surface of the metal substrate, ME-SOG system does not show unique distance-dependent behavior. For example, Zhang et al. [17] observed that the ME-SOG of Rose Bengal has achieved the maximum enhancement when there is no spacer between the Rose Bengal molecules and the surface of silver island film. However, when silica spacer was introduced, the enhancement factor decreased gradually when the thickness of silica spacer is increased. They also have correlated this trend of enhancement to the enhanced electric filed around the silver island film, which decreased exponentially into the surrounding. In another study, Hu et al. [181] reported that the maximum ME-SOG of Al(III) phthalocyanine chloride tetrasulfonic acid (AlPcS_4_) molecules occurs at a certain distance from the surface of Au nanoring fabricated on glass substrate, where the distance is well controlled by the LBL approach using Poly(allylamine hydrochloride)/poly(sodium 4-styrenesulfonate) (PAH/PSS) bilayers. They have observed that this optimum distance depends on the aspect ratio of Au nanorings (Fig. 11b). The latter indicates that the type and shapes of metal nanoparticle could be a key factor in ME-SOG due to the different enhanced electric field as well as different rates of energy transfer from the excited PS molecules to the surface of metal nanostructures.

Besides fundamental studies, the practical application of ME-SOG in the planar metal-PS system has also been explored. For example, silicone has been used as a flexible substrate to replace rigid planar substrates such as glass or quartz for antibacterial applications. In one study, Noimark et al. [182] embedded the gold NPs into the silicone polymers first, followed by immersing the silicone polymer into the crystal violet solution to load the molecules on AuNPs. The final nanocomposite showed very effective antibacterial properties against *Staphylococcus epidermidis* and *Escherichia coli* as well as inhibited the formation of *Staphylococcus epidermidis* biofilms [183]. In a separate study, Perni et al. [184] reported that by incorporating Methylene Blue as photosensitizer and 2 nm AuNPs in polysiloxane film, there was 99.97% reduction in the counts of methicillin-resistant *Staphylococcus aureus* and *Escherichia coli* with only 5 min irradiation of 660 nm laser light. In addition, they have studied the effect of the size of AuNPs (2, 5, and 20 nm) on the antibacterial properties of Methylene Blue containing silicone film [185]. It was found that the 2 nm AuNPs could enhance the antibacterial properties while no effects were observed on those bigger AuNPs containing film. Even worse, these larger AuNPs had some shielding effect, which affect the SOG and consequently the antibacterial performance. It should be mentioned that in this study, the same molar concentration of gold salts has been used for the synthesis of AuNPs of different sizes. The amount of smaller AuNPs (i.e., 2 nm) was much more than the larger ones (i.e., 5 nm and 20 nm AuNPs), meaning that the inter-particle distance was smaller (more crowded) between those smaller AuNPs. This effect might be overlooked as the electric field around metal NPs could be enhanced dramatically when the inter-particle distance is in nm range due to the formation of hot-spots in the electric field distribution around the metal NPs [186, 187]. Consequently, the excitation rate of the photosensitizer molecules was enhanced in the vicinity of the metal NPs [17]. In addition to silicone, polyurethane also has been used to embed the AuNPs and different PS molecules like Methylene Blue and toluidine blue for antibacterial study [188]. It has been shown that *Staphylococcus aureus* were killed at about 2.8log_10_ (i.e., 99.84%) and 4.3log_10_ (i.e., 99.995%) by applying white light illumination on the film containing Au NPs@MB and Au NPs@TBO, respectively.

### Metal-Enhanced Singlet Oxygen Generation in Colloidal Systems

As discussed earlier, the ME-SOG in the metal-PS planar system was commonly used for antibacterial applications and not suitable for in vivo applications due to geometric limitations. ME-SOG in the colloidal metal-PS systems is more suitable for in vivo applications such as cancer treatment. In addition, the colloidal systems can be more flexible, allowing combination of other therapeutic methods like drug delivery and photothermal therapy.

**Fig. 12:**
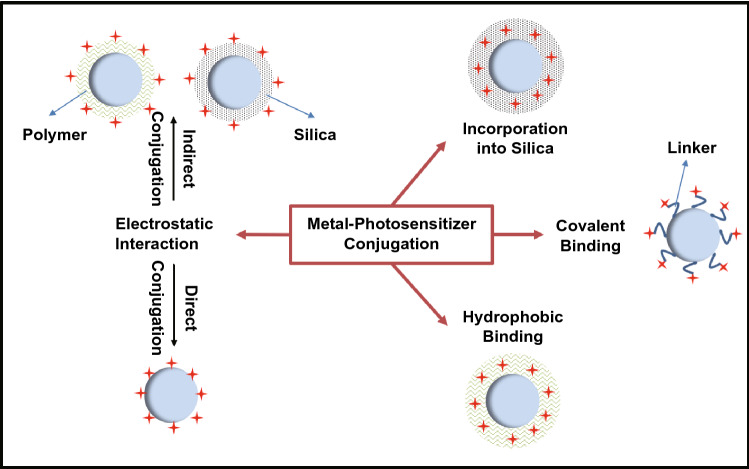
Conjugation methods of photosensitizers molecules to the metal nanoparticles in forming colloidal metal-PS system for metal-enhanced singlet oxygen generation

The first consideration for the design of the ME-SOG in colloidal system is the surface property of metal nanoparticles (NPs). Colloidal metal NPs are usually covered with organic molecules as the capping or passivating agents to protect the colloidal NPs from aggregation and remain stable in the solution. These capping agents ranging from small molecules like citrate to long chain polymers. Sometimes, a dielectric layer such as silica or polymer could be introduced to control the distance between the metal NPs and photosensitizer molecule. Both the capping agent and/or dielectric layer can play an important role in affecting the ME-SOG in colloidal metal-PS systems. As is shown in Fig. 12, both polymer and silica can be used to load the photosensitizers. Some photosensitizers can bind directly to the surface of dielectrics or metal NPs via electrostatic interaction. In this section, we review the different approaches for conjugating photosensitizers and metal NPs to form the colloidal metal-PS system, follow by the discussion about the different effective factors contributing to the ME-SOG performance.

#### Colloidal Metal-Photosensitizer Systems without Additional Spacer

**Fig. 13:**
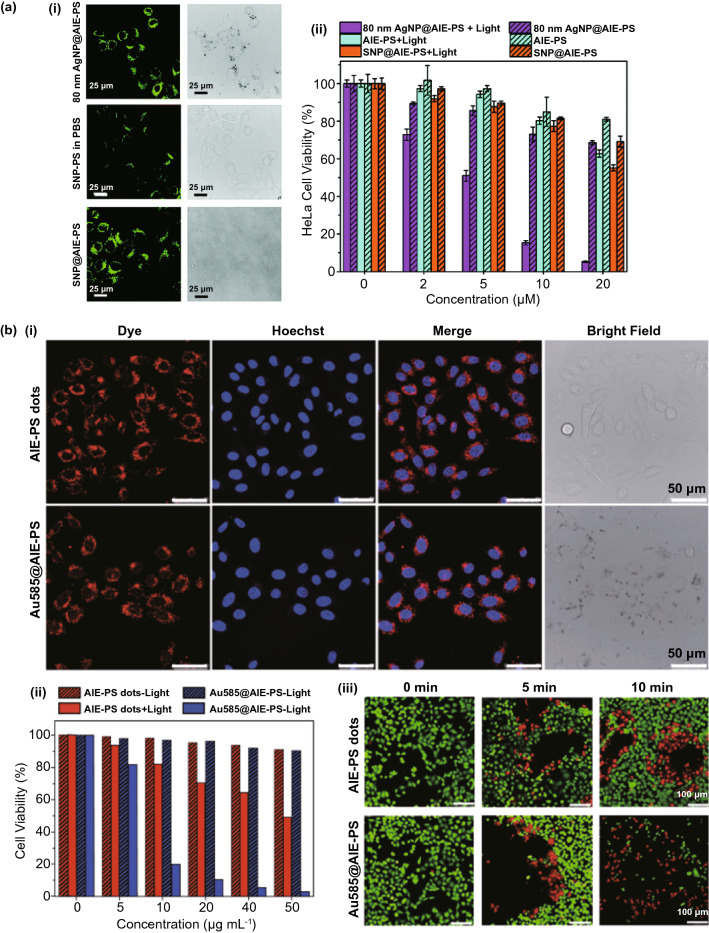
**a** (i) Confocal fluorescence imaging and bright field images of HeLa cells incubated with 80 nm AgNP@AIE-PS (top row) and AIE-PS dots (bottom row) in physiological buffer solution (PBS), respectively. Silica nanoparticles (SNP) @AIE-PS is used as the control samples, (ii) cytotoxicity of the same samples in **a** under white light illumination and at darkness against HeLa cells, reproduced from Ref. [22] with permission from Royal Society of Chemistry, **b** (i) Confocal fluorescence imaging and bright field images of HeLa cells incubated with AIE-Ps dots and Au585@AIE-PS dots samples. The nuclei were stained with Hoechst dye., (ii) Cell viability of HeLa cells after incubation with different concentrations of AIE-PS and Au585@AIE-PS samples with and without white light illumination. (iii) Live/dead cell staining of HeLa cells before (0 min) and after light treatment for 5 and 10 min, respectively, reproduced from Ref. [21] with permission from Springer-Nature

The interaction between the metal NPs and PS molecules is an important factor in ME-SOG study. Photosensitizer molecules can be attached directly to the metal NPs surface with their capping agent through either electrostatic interaction or covalent binding. Some examples on how the metal NPs with various capping agents are attached to the different photosensitizer molecules are listed in Table 2. These metal NPs are mostly spherical gold and silver (AuNPs and AgNPs) due to their unique optical properties and well-established synthesis methods. For the PS molecules, porphyrins and phthalocyanines (Pc) are mostly studied in ME-SOG as they have been clinically approved for their efficiency in ROS generation. Furthermore, their optical properties, especially their absorbance spectra that is located in the visible to the NIR range, can interact well with the plasmonic nanoparticles [189]. Different derivatives of phthalocyanines have been functionalized with thiol group to facilitate their conjugation with the metal NPs, e.g., AuNPs via the Au–S bonding [190, 191]. There are also a few reports on the use of electrostatic interaction to load the phthalocyanines on metal NPs surface [192, 193]. For other example, Zhou et al. [192] synthesized a water-soluble dendritic carboxylated-zinc phthalocyanine as photosensitizer with no fluorescence observed in water. However, the fluorescence can be turned on when it was electrostatically adsorbed onto the surface of cetrimonium bromide-capped gold nanorods (AuNRs) due to the MEF effect. In addition, the SOG rate was enhanced about 2-fold due to the plasmonic effect of AuNRs. In another study, Li et al. [193] have reported the simultaneous SOG and fluorescence enhancement of sulfonated-AlPc after adsorption onto the surface of cetrimonium bromide-capped AuNRs. The AuNR-PS conjugates can penetrate the QGY liver cancer cells (human hepatocellular carcinoma cell line 7701) for dual modal PDT and photothermal therapy as well as bioimaging. Recently, we have studied the correlation between MEF and ME-SOG in AgNPs enhanced AIE photosensitizer (PS) system, including NP size and Ag-PS distance effects [20]. The distance between the AgNP core and AIE-PS was carefully tuned by changing the number of polyelectrolyte bilayers consisting of poly ethyleneimine and poly(styrene sulfuric acid) sodium salt via LBL deposition. It was observed that ME-SOG occurred even when no polyelectrolyte is deposited on surface of AgNPs, while larger AgNPs resulted in higher ME-SOG rate. The concentration and size effects of core AgNPs is also investigated on SOG enhancement using a positively charged red-emissive AIE photosensitizer as a model system [22]. We found that the ME-SOG achieved the maximum when specific concentration of AgNPs is attached directly to the AIE photosensitizer molecule without additional spacer. It was found that the optimum concentration of AgNPs for maximum SOG enhancement was inversely proportional to the size of AgNPs. The as-developed AgNP@AIE photosensitizer in this study was used for simultaneous fluorescence imaging and photodynamic ablation of cancerous HeLa cells (Fig. 13a). In another study, we used Au nanostars (NS) and AIE photosensitizer formulated nanodots to develop the highly effective fluorescent AuNS-AIE nano-photosensitizer exhibiting both MEF (10% fluorescence enhancement) and ME-SOG (a maximum of 15-fold singlet oxygen generation enhancement) for image-guided PDT of cancerous HeLa cells (Fig. 13b) [21].

**Table 2:** Colloidal metal-photosensitizer systems without additional spacer

Type of metal NPs (size)	Capping agent	Photosensitizer	ROS EF/Triplet EF	attachment method	Other Applications	Refs.
Ag NPs (5.4 nm)	Glutathione	ZnPc	2.19 Singlet oxygen	Covalent bonding	Antibacterial against *E-Coli*	[195]
Ag NPs (19, 66, 106 nm)	PEI	PpIX	2.56-fold, 4.72-fold	Electrostatic interaction	Size-dependent Comparison of cell uptake and intracellular ROS generation with cell-free data	[205]
-Ag NPs (9 nm)	Pectin	Riboflavin	Singlet O: 30% hydrogen peroxide: 60%	Electrostatic interaction	–	[200]
Ag NPs (5.3 nm)	1-nonanthiol	Chl	2-fold	Electrostatic interaction	–	[199]
Au NPs (2–4 nm)	Zn- phthalocyanine and TOAB	ZnPc	44% Singlet oxygen phosphorescence QY	Au–S bonds	–	[206]
Au NPs (5.3 nm)	TOAB	ZnPc	1.51-fold	Au–S bonds	–	[207]
Au NPs (5 nm)	TOAB	Three complexes of ZnPcs	Both enhancement and quench for triplet QY	Au–S bonds	–	[208]
Au NPs (5.37 nm)	TOAB	ZnPc	–	Au–S bonds	PDT for MCF-7 cells	[197]
Au NPs (5 nm)	PEG and Zn phthalocyanine	ZnPc	–	Au–S bonds	PDT for SK-Br-3 cells	[198]
Au NPs (15 nm)	ATP	MB Azure Thionine	Enhanced triplet state	Electrostatic	–	[209]
Au NPs (5.4 nm)	CTAB	AlPc	1.75-fold	Electrostatic	–	[210]
Au NPs (25 nm)	Cysteamine	RB	13-fold (based on Phosphorescence of singlet oxygen)	Covalent bonding	–	[211]
Au NPs (15 nm)	PEG5000	Pheophorbide Hematoporphyrin	10% 22%	Electrostatic interaction	–	[212]
Au NPs, aggregated (40–50 nm)	PEI or Citrate	PpIX	4-fold	Electrostatic interaction	PDT for Breast cancer cells	[204]
Aggregated Au NPs (54 nm)	Pluronic F127	MB	About 2-fold	Electrostatic interaction	Murine colon carcinoma cells (C-26) Imaging SERRS FLIM	[203]
Au NRs (AR: 2.1)	CTAB	dendritic ZnPc	2 singlet oxygen	Electrostatic interaction	–	[192]
Au NRs (AR: 3.7) Au bipyramids (AR: 2.9)	CTAB	AlPc	1.96-fold 2-fold	Au–S bonds	Antifungal against C. albicans Antibacterial against E-Coli	[196]
Au NRs (AR: 3.6)	CTAB	Sulfonated AlPc	–	Electrostatic interaction	PDT and PTT for QGY cells	[193]
Au NRs (61 × 16 nm)	CTAB	Two ZnPcs	3-fold	Electrostatic interaction	PDT for Melanoma Cells	[213]
Au NRs (3.2 AR) Au pyramids (AR:2.5)	CTAB	AlPc	1.96-fold 2-fold	Electrostatic interaction	50% quench of PL	[210]
Au NRs (40 × 10 nm) Au NPs (40 nm) Ag NPs (40 nm)	Mercaptoethylamine m-PEG114	PpIX	Different amount From 2- to 13-fold	Electrostatic interaction or Covalent bonding	–	[214]
Au NRs/(50 nm length, AR: 4.2)	CTAB	Ce6	1.3-fold	Electrostatic interaction	PDT of Hella cells and KB Cells	[215]
Au NPs (20 nm)	Cysteine	Protoporphyrin IX	0.2-fold	Covalent bonding	–	[216]
Hollow gold nanospheres (HAuNS) (50 nm)	PEI	ICG	2.6-fold	Covalent bonding	PTT and PDT	[217]
Au Nanoring (100 × 50 nm)	PEG	AlPcS	2.9-fold TP	Covalent bonding	PTT and PDT	[218]
Au bipyramids (AR: 3) Au nanorods (AR: ~ 5)	CTAB	AlPcS	11.5-fold TP 2.1-fold TP	Electrostatic interaction	In vivo two photon PDT	[219]
Au nanoparticle (4–6 nm)	–	Carbon dots (tannic acid and polyethyleneimine)	2.3-fold	Nanocomposite	–	[220]

*CTAB* cetyl trimethylammonium bromide, *PEG* poly(ethylene glycol), *TOAB* tetraoctylammonium bromide, *ATP* adenosine triphosphate; *PEI* polyethyleneimine, *Pp* protoporphyrin, *MB* methylene blue, *RB* rose bengal; *Pc* phthalocyanines; *Chl* chlorophyll, *TP* two photons, *PTT* photothermal therapy

For the covalent attachment, thiol-functionalized phthalocyanine (thiolated-Pc) is most commonly used to bind with the surface of metal NPs due to the high stability of the final conjugates in biological media and relatively low chance of undesired leakage of thiolated-Pc molecules. Two different approaches can be used to conjugate the thiolated-Pcs molecules to metal NPs. The first method is a direct synthesis of phthalocyanine-capped AuNPs as developed by Brust et al. [194] It is based on the phase transition approach where the phthalocyanine molecules are dissolved in an organic solvent containing Tetraoctylammonium bromide, while the Au precursor is dissolved in water. Briefly, the two phases are first mixed to allow the transfer of Au ions from the aqueous phase to organic phase. Then, then extra aqueous phase is removed followed by the addition of a reducing agent (e.g., NaBH_4_). The final product is phthalocyanine/TOAB-capped AuNPs, which are soluble and stable in organic solvents. The other method is similar to the ligand-exchange method, where the AuNPs are first synthesized first, followed by the attachment of thiolated-phthalocyanine molecules via the strong Au–S bond. The latter approach is much more flexible as compared the Brust’s method, as the AuNPs can be synthesized and modified via different chemistry. Furthermore, the final product possesses both thiolated-phthalocyanine and water-soluble capping agent on the metal NPs surface, which allow them for use in different biological applications.

Zinc phthalocyanine derivatives are the most commonly used phthalocyanines to form the metal- phthalocyanine conjugates. Rapulenyane et al. [195] have used the glutathione-capped Ag NPs (5.4 nm) to conjugate with Zn–Pc molecules. It has been reported that the final conjugates were able to kill the *E-Coli* even at a low concentration range of 5–10 μM, which could not be achieved by ZnPc alone. This observation was attributed to the enhanced singlet oxygen generation of about 2.19-fold when the ZnPc was attached to the Ag NPs. In another study [196], anisotropic Au nanostructures were used to study their effect on SOG of Aluminum-Phthalocyanine derivate (AlPc). It was observed that both AuNRs (aspect ratio of 3.7) and Au bipyramids (aspect ratio of 2.9) could enhance the SOG of AlPc at about 1.96-fold and 2-fold, respectively. The final conjugates were also used as an antibacterial and antifungal agent against *E-Coli* and *C. albicans* where 3.71log_10_ (99.98%) and 2.53log_10_ (99.70%) reduction were observed, respectively. Besides, the AuNPs-ZnPc conjugates have also been used for photodynamic therapy of cancerous cells, which showed promising toxicity when they were illuminated by incident light during PDT [197, 198]. However, Nombona et al. [197] reported that the liposome containing the Zn-Pc molecules were more efficient than Au-ZnPc conjugate with the same amount of photosensitizer.

As phthalocyanines are mostly insoluble in water, this has driven the development of water-soluble photosensitizers to allow easy conjugation with the metal NPs either via covalent chemical bonding or electrostatic interaction in aqueous solution. As most of PS molecules have a carboxylic group, a positive surface charge is needed for metal NPs to interact with PS molecules electrostatically. CTAB and cationic polymers are the most frequently used capping agents used to synthesize metal NPs with positive surface charge. These photosensitizer molecules also allow covalent conjugation with the metal NP (e.g., EDC/NHS coupling) due to the presence of functional groups (especially carboxyl group) on their surface. For example, Bekalé et al. [199] have studied the hybrid system composing of Chlorophyll which was adsorbed on the 1-nonanthiol-capped Ag NPs (5.3 nm) electrostatically. The chlorophyll-AgNPs has resulted in 2-fold enhancement in singlet oxygen generation rate and 20% fluorescence enhancement as compared to the Chlorophyll PS molecules alone. In another study [200], it has been shown that the generation of both singlet oxygen and hydrogen peroxide by Riboflavin can be enhanced by about 30% and 60%, respectively, when Riboflavin is conjugated to pectin-capped AgNPs.

As introduced previously, metal-enhanced SOG is based on an enhanced excitation rate in the PS molecule as a result of the enhanced electric field around the metal nanostructures. Hence, designing a new metal nanostructure such as anisotropic bipyramid nanosilver [201] or dimeric silver nanocubes [202] to enhance the electric field is an excellent approach to promote the singlet oxygen generation. It has been well studied and shown that aggregated metal nanoparticles could result in the enhanced electric field and form the plasmonic “hot-spot” to enhance the singlet oxygen generation by photosensitizer molecules. For example, Simon et al. [203] have synthesized aggregated Au NPs by adding NaCl solution to the Pluronic-capped spherical Au NPs in methanol. In this approach, methanol plays an important role as it is less polar than water, which can facilitate the aggregation process by reducing the electrostatic repulsive force between Pluronic-capped AuNPs. Finally, Methylene Blue molecules were added to the Pluronic block copolymer-capped aggregated AuNPs (Au nanoaggregates). The results showed about 2-fold enhancement in SOG production. Moreover, the obtained Au nanoaggregates were applied for imaging of murine colon carcinoma cells (C-26) using different techniques including scanning confocal surface-enhanced resonance Raman spectroscopy (SERRS) and fluorescence lifetime imaging. In another study by Yang et al. [204], the intracellular aggregation method has been used to develop AuNP-PpIX nanohybrid for enhanced PDT. In this method, breast cancer cells were incubated with both the positively charged AuNPs and negatively charged AuNPs loaded with PpIX as a photosensitizer. It has been shown that the PDT enhancement factor is 4-fold which could be due to the intracellular aggregation of oppositely charged AuNPs with to the formation of plasmonic hot-spots for SOG enhancement.

#### Colloidal Metal-Photosensitizer Systems with Dielectric Spacer

Same as the metal-photosensitizer planar systems, ME-SOG in the colloidal systems are also distance-dependent. Thus, different types of dielectric spacer are introduced to control the distance between the photosensitizer molecules and metal NPs. Silica and polymeric spacers are the two most popular dielectric spacers being developed. As shown in Fig. 12, photosensitizer molecules can be attached to the surface of dielectric electrostatically, covalently and/or embedded inside the dielectric. When the photosensitizer molecules are embedded inside the dielectric layer such as silica, the final nanostructure is more biocompatible (due to the inertness of the dielectric layer) and has more room for further modification (e.g., the mesoporous structure of silica shell) for In vivo applications with less dark cytotoxicity as the photosensitizer molecules are not directly exposed. When the photosensitizer molecules are attached on the surface of dielectric layer through electrostatic interaction or covalent binding, the distance between the photosensitizer and metal nanoparticles are relatively easier to control.

##### Silica Shell as Spacer

**Fig. 14:**
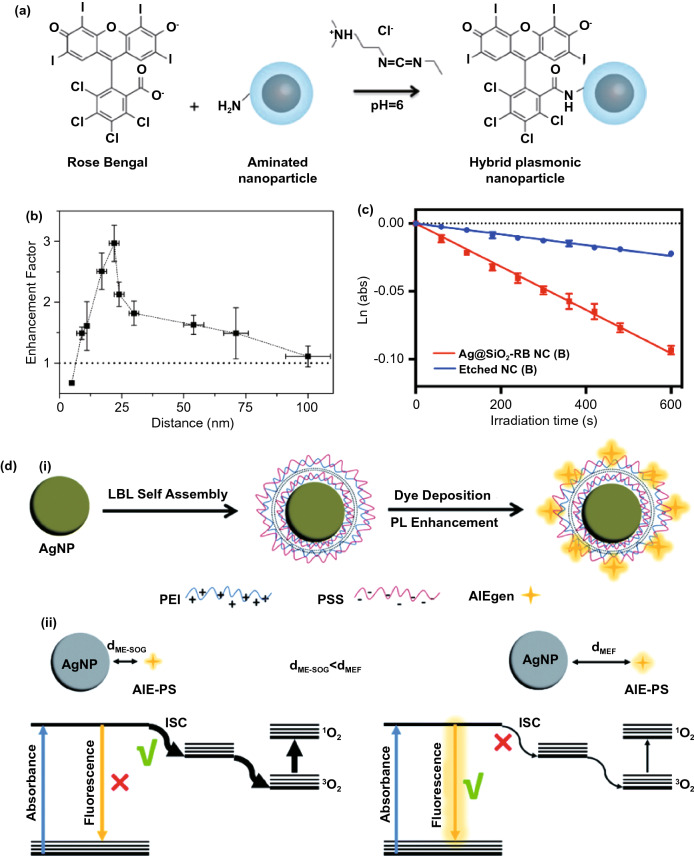
**a** Chemical bonding of Rose Bengal to aminated metal@silica nanoparticle, reproduced from Ref. [223] with permission from American Chemical Society. **b** Distance-dependent ME-SOG of Rose Bengal on 67 nm Ag nanoparticle coated with silica shell, reproduced from Ref. [223] with permission from American Chemical Society. **c** The first-order kinetic data for degradation of ABDA molecules in the presence of Rose Bengal molecules bonded on silver nanocube coated with silica shell (Ag@SiO_2_-RB NC) and the etched sample as control, and under similar white light irradiation, reproduced from Ref. [224] with permission from American Chemical Society. **d** (i) Layer-by-layer approach to control the distance between metal core and photosensitizer by tuning the thickness of dielectric bilayer on metal nanoparticles surface. (ii) Simplified Jablonski diagrams showing the MEF (right scheme) and ME-SOG (left scheme) mechanism of the AIE-PS nanohybrids occur at different optimum AgNPs to AIE-PS distance for maximum plasmonic enhancement, reproduced from Ref. [20] with permission from Royal Society of Chemistry

Silica shell is one of the most popular spacers in colloidal ME-SOG systems due to its well-established synthesis protocol and good optical transparency. Moreover, silica shell is usually porous, which allows the adsorption of PS molecules onto its surface and inside the pores. For example, Jijie et al. [221] have reported a 3.5-fold SOG enhancement using indocyanine green as PS and Au nanorods@silica (i.e., Au NRs with 3.4 aspect ratio and silica thickness of 20 nm). The as-developed colloidal ME-SOG system have been used for the inactivation of a Crohn’s disease-associated Escherichia coli strain. In addition, the silica surface can be modified with alkylo-silane containing different functional groups that enable the covalent attachment of PS molecules on its surface or even inside the holes of the silica shell. For example, Mooi and Heyne [222] have synthesized Ag@silica@Rose Bengal via covalent attachment of Rose Bengal molecules to the amine-functionalized Ag@silica NPs containing 68 nm Ag core and 25 nm silica thickness (Fig. 14a) to achieve a 3.8-fold enhancement in SOG. A similar study by Planas et al. [223] reported a 3-fold enhancement of SOG by Rose Bengal by using 67 nm Ag NPs core with 11 nm silica shell thickness (Fig. 14b). The SOG can be further enhanced by using anisotropic metal nanoparticle like silver nanocubes, where 4-fold SOG enhancement has been reported for Ag nanocubes (44 nm) and Rose Bengal with a separation distance of 10 nm [224] (Fig. 14c). The group has shown that scattering of the metal core plays an important role in ME-SOG. They have observed that AgNPs exhibited higher ME-SOG than the AuAgNPs and/or AuNPs in the same metal@silica@Rose Bengal systems [225, 226]. To enhance the local concentration of photosensitizer molecules, Rosa-Pardo et al. [227] have grown mesoporous silica shell on metal NPs and then, attached the Rose Bengal molecules inside the functionalized silica pores via covalent binding where 1.5-fold SOG enhancement have been found in this system. On the other hand, PS molecules can be added to the reaction vessel during the synthesis of silica to form the PS embedded silica. For instance, Zhao et al. [228] have developed the AuNRs@silica NPs containing hematoporphyrin PS molecules which a 20% enhancement in SOG. In another study, AuNRs@silica containing ICG have been used as an efficient simultaneous PTT and PDT agent with about 3.6-fold PDT enhancement [229].

Instead of coating a silica layer on the surface of metal nanoparticles, some researchers deposit metal nanoparticles on the preformed silica NPs. Zampini et al. [230] have studied the effect of the addition of Au NPs to the solution of silica NPs doped with PpIX. They have reported a 2.9-fold enhancement in SOG due to the interaction of cysteamine-capped AuNPs with silica-doped PpIX NPs. Besides porphyrin-based photosensitizers, other photosensitizers such as methylene blue have been used for ME-SOG related applications [231]. Methylene blue showed different behaviors in generating singlet oxygen and other reactive oxygen species in a solution or localized position when attached to or adsorbed on the surface of solid materials like silica. Therefore, Chu et al. [232] has studied the ME-SOG of methylene blue using AuNR@silica that contains methylene blue inside the silica shell. It is found that all three types of ROS including singlet oxygen, superoxide and hydroxyl oxide were generated by the methylene blue molecules and were enhanced due to the plasmonic effect of AuNRs. It should be noted that methylene blue can mostly generate singlet oxygen in solution, while when its local concentration increases (for example inside the silica shell), it would be able to generate different types of ROS as reported by Baptista and colleagues [233].

##### Polymeric Layer as Spacer

Polymeric spacer is of interest for biological applications due to their biocompatibility and other properties like stimuli-responsivity. Different layers of polymers can be deposited on the surface of colloidal nanoparticles based on the layer-by-layer assembly of oppositely charged polyelectrolytes in solution. For example, we have used the layer-by-layer approach to investigate the correlation between the MEF and ME-SOG in a metal-AIE photosensitizer colloidal system. We found that maximum ME-SOG occurs at a distance much shorter than that required in achieving highest MEF (Fig. 14d), which is an important parameter for designing dual-functional (i.e., fluorescence and SOG ability) metal-enhanced systems for image-guided PDT. Besides, the polymer shell can be grown around the metal NPs via polymerization approach. Similar to the use of silica as a spacer, photosensitizer molecule can be adsorbed on the surface of the polymeric shell electrostatically/covalently/entrapped inside the polymeric shell during the polymerization process. As can be seen in Table 3, different types of biocompatible polymers have been used as the spacers for ME-SOG study.

**Table 3:** Colloidal metal-photosensitizer systems with different types of dielectric spacer

Metal NPs (size)	Spacer/Thickness	Photosensitizer	ROS EF	Attachment method	Applications	Refs.
Ag NPs (68 nm)	Silica/25 nm	RB	3.8-fold	Covalent bonding	–	[222]
Ag NPs (67 nm)	Silica/11 nm	RB	3-fold	Covalent bonding	–	[223]
Ag NPs	Glycopolymer	BODIPY	3-fold	Covalent bonding	PDT against P. aeruginosa, S. aureus and NIH3T3 cells	[234]
Ag NPs (< 10 nm)	Silica/10 nm	β-NaYF4:Nd/Yb/Ho	–	Covalent bonding	–	[235]
Ag Nanocube (44 nm)	Silica/10 nm	Rose Bengal	4-fold	Covalent bonding	Bacterial inactivation	[224]
Ag NPs (15 nm) Au NPs (15 nm) AgAu NPs (15 nm)	Glutathione	Two ZnPcs (ZnPc(1) and ZnPc(2))	ZnPc(1): 14% ZnPc(2): 25% ZnPc(1): 23% ZnPc(2): 30% ZnPc(1): − 17% ZnPc(2): − 16%	Covalent bonding	–	[236]
Au NRs (35*9.3 nm, AR: 3.8)	Polyacrylic acid (PAA)	Toluidine Blue O (TBO)	1.15-fold	Covalent bonding	Bactria killing	[222]
Au NRs (46.8*19.4 nm)	Silica/10.6 nm	tetra-substituted carboxyl AlC4Pc	2.1-fold	Covalent bonding	–	[237]
Au NRs (AR: 4.25)	Silica/20 nm	(T790)	Quench (One-photon) 7.6-fold (Two-photons)	Covalent bonding	Lifetime PDT	[238]
Au NRs(AR: 3.47)	Silica/21 nm	ICG	1.77-fold	Covalent bonding	In vitro PDT	[239]
Fe3O4@Au (5 nm/3 nm)	Silica/embedded	RB	1.5 times	Covalent in pores	–	[227]
Ag NPs (25 nm)	Silica	HPIX	3.5-fold based on phosphorescence	Embedded in silica	Broadening the absorbance spectra PDRT of Hela Cells	[240]
Au NPs (4.6 nm)	Silica	Protoporphyrin IX	2.3-fold	Embedding in Silica	–	[230]
Au NRs (49 × 14.4)	Silica/17.6 nm loaded PS	ICG	3.6-fold	Embedded in Silica	PTT and PDT Single laser	[229]
Au NRs, (45 nm × 15 nm AR:3)	Silica/embedded /10 nm	Hematoporphyrin	1.2-fold two-photon	Embedded in Silica	Two-photon imaging	[228]
Au NRs (AR: 3–4)	Silica	MB	–	Embedded in Silica	Enhanced in vivo therapy	[231]
Au NRs (AR = 2.3)	Silica	MB	1.16 via upconversion excitation	Embedded in silica	–	[241]
Au NRs (AR: 2)	Silica/40 nm	MB	Competition between singlet and superoxide	Embedded in Silica	PDT of HepG2 cells	[232]
Au NRs (AR: 3.4)	Silica/20 nm	Pd-meso-tetra(4-carboxyphenyl) porphyrin (PdTPP)	4-fold two photon	Embedded in Silica	PDT + PTT	[242]
Au Nanostar (70 nm)	Silica	MB	50%	Embedded in silica	SERS PDT of BT549 cells	[243]
Ag NPs (52 nm)	PLGA	Hypocrellin B	About 2-fold	Embedding in PLGA	Polymer NP contains both PS and Ag NPs MEF and ME-SOG	[244]
Ag NPs (5–25 nm)	Polymethyl-methacrylate	Porphyrin	Not mentioned	Embedded in polymer	Enhanced antibacterial properties	[245]
Ag NPs (13 nm)	Pectin	Riboflavin	1.8-fold	Electrostatic interaction	–	[246]
Ag NPs (16 nm)	Chitosan	Curcumin	1.5-fold	Electrostatic interaction	–	[247]
Ag NPs (60 nm) Ag NPs (100 nm)	Silica/6 nm	Protoporphyrin IX-	1.9-fold 5.4-fold	Electrostatic interaction	–	[248]
Ag NPs/42 nm	Silica/2 nm	HPIX	3 to 4-fold	Electrostatic interaction	Antibacterial: 6-fold	[249]
Au NPs (21 nm)	polystyrene-altmaleic acid (PSMA)/10 nm	MB	1.25-fold	Electrostatic interaction	–	[250]
Au NPs (8 nm)	CeF3	Verteporfin	1.64-fold	Electrostatic interaction	–	[251]
Au NRs(35 × 9.3 nm; AR: 3.8)	PEI PSMA	ICG	1.8-fold	Electrostatic interaction	PDT, PTT	[252]
Au NRs (98 × 46 nm)	CTAB/PSS	TiO2	2.3-fold	Electrostatic interaction	–	[253]
Au NRs (52*13 nm)	CTAB/PAH	Rose Bengal	1.1-fold	Electrostatic interaction	Effect of light color	[254]
Au NRs (AR = 6)	Silica/embedded	ZnPc	6 to 7-fold via upconversion excitation	Electrostatic interaction	Conjugation with upconversion nanoparticle PDT + PTT	[255]
Au NRs (AR = 3.5)	Silica/20 nm	Verteporfin	1.2-fold	Electrostatic interaction	–	[256]
Au NRs (AR: 3.4)	Silica/20 nm	ICG	3 to 4-fold	Electrostatic interaction	Inactivation of a Crohn’s disease-associated Escherichia coli strain	[221]
Au NRs (AR = 2.86)	Silica	Merocyanine 540	1.6-fold via upconversion excitation	Electrostatic interaction	Fluorescence imaging Using upconversion nanomaterial The single wavelength for PTT and PDT	[257]
Multi-branched Au NPs/about 50 nm	Silica + LBL of PLL/PAA	Por4 +	2-fold	Electrostatic interaction	Antibacterial against E.Coli	[258]
Au Nanostar (100 nm)	Silica	ZnPc	2-fold via upconversion excitation	Hybridization of DNAs	PDT + PTT	[259]
Au NPs (40 nm)	poly(NIPAAm- b-styrene)/30 nm	Ce6	2-fold based on Phosphorescence	Hydrophobic–hydrophobic interaction	Enhanced antibacterial against Staphylococcus aureus	[260]

*T790* trimethoxyl(octadecyl)silane (OTMS), mesotetra(4-carboxyphenyl) porphyrin

#### Critical Parameters on Metal-Enhanced Singlet Oxygen Generation

Designing a perfect metal-photosensitizer nanohybrid system with maximum PDT efficacy relies on the true understanding of metal-enhanced singlet oxygen generation (ME-SOG) system and the effective parameters on such a complex system. In this section, we review the parameters of metal nanostructure (e.g., size, shape and composition) and photosensitizer (e.g., quantum yield, excitation and emission wavelengths) that can determine the performance of a ME-SOG system. Additionally, we discuss how ME-SOG system can affect other optical properties of the photosensitizer molecule, which lead to better performance of the photosensitizer in the nanohybrid system.

##### Size, Shape and Composition of Metal Nanoparticles

In any PS molecule, the rate of ROS production is maximum when the photosensitizer molecule absorbs more energy. This occurs when the incident light is well matched with the maximum absorbance peak of photosensitizer molecule (*λ*_max_). Hence, PDT would be more efficient when the photosensitizer molecule is excited by the light source with the same wavelength as *λ*_max_. In this case, the spectral overlap between the extinction of metal nanostructure and the absorbance of photosensitizer molecule is important due to the resonance between the plasmonic NPs and the excited photosensitizer molecules. Hence, it is expected that a higher spectral overlap would lead to a higher SOG enhancement. Systematic studies on the use of AuNPs and different photosensitizers have shown that wider spectral overlap would result in producing more triplet-excited states [209]. Furthermore, different works have shown that spectral overlap plays an important role in MEF and energy transfer between the excited molecule and metal, which is very similar to ME-SOG [261, 262]. Based on Mie theory, the plasmonic properties of metal NPs is a function of composition, shape, and size of NPs. Thus, the degree of spectral overlap is controlled by choosing the right size, shape and composition of metal core, which determine its surface plasmon band. ME-SOG phenomena are also a consequence of plasmonic effects as well as the interaction between the excited PS molecules and metal NPs. Thus, the size, type, and shape of metal NPs also play a vital role in ROS enhancement as they could directly affect the electric field enhancement around the metal NPs as well as extinction spectra of metal NPs.

Ag and Au nanostructures are the two most widely used metal nanostructures for ME-SOG due to their well-developed synthesis protocols, high colloidal stability and more importantly, strong and tunable absorptions in the visible to NIR or even IR range. The spectrum of Ag and Au nanostructures can be classified into two main groups according to their unique LSPR properties: 400–650 nm for Ag nanostructures and 500–1000 nm for Au nanostructures. Hence, the selection of the type of metal nanostructure mostly depends on the absorbance spectra of the photosensitizer. In the range of 500–650 and even 700 nm, both individual Au and Ag NPs, as well as alloy AgAu nanostructures can be used. In the study by Dube et al. [236], the effect of spherical Au NPs (*d* = 13.6 nm, *λ*_max_ = 532 nm), Ag (*d* = 11.1 nm, *λ*_max_ = 405 nm) and AuAg NPs (*d* = 15.1 nm, *λ*_max_ = 408 nm) on SOG by two different complexes of ZnPcs (with *λ*_max_ = 675 nm and 680 nm) were investigated. It was observed that Au NPs led to more enhancement of SOG than AgNPs because of the large overlap between the extinction of AuNPs and PS molecules, while AuAgNPs resulted in reduction of SOG ability, which could be due to screening effect [263] or the intrinsic properties of AuAg alloy NPs. In another study, Macia et al. [264] have shown that spherical AgNPs could enhance the SOG produced by Rose Bengal more than the AuNPs and AuAgNPs with similar size in a metal@silica@RB colloidal system. Based on their experimental studies and simulation results, it is concluded that a stronger scattering by AgNPs would provide more light to be absorbed by the RB molecules, and thus a higher SOG rate was observed for AgNPs@silica@RB hybrid sample. More recently, we have synthesized five different Au nanostars (i.e., Au540, Au585, Au668, Au718, and Au762) to tune their spectral overlap with the fluorescence spectra of a red-emissive AIE-PS nanodots [21]. It was observed that the scattering efficiency is not the only parameter that determines the ME-SOG, where a combination of enhanced electric field as well as scattering efficiency and non-radiative energy transfer rate from excited AIE-PS to Au nanostar results in a complex system. These results show that ME-SOG system behaves nonlinearly and more fundamental studies on the design parameters of metal/photosensitizer pairs would need to be conducted for understanding of the complex ME-SOG system.

The size effect of different metal nanoparticles has been investigated by several groups. For example, Lismont et al. [248] synthesized Ag@Silica@PpIX system and studied how SOG of the Pplx photosensitizer was affected by varying the core size of AgNP. The 100 nm AgNPs were found to enhance SOG of PpIX by about 5 times while the 60 nm AgNPs were not able to improve the SOG. This could be due to a larger spectral overlap between the extinction of 100 nm Ag NPs with the absorbance of PpIX as compared to that of the 60 nm Ag NPs. The results for the size effect of metal core on ME-SOG in this study was consistent with the MEF results, where the 100 nm AgNPs were able to enhance the fluorescence of PpIX much more than the 60 nm AgNPs. In another work, the effect of AuNPs of different sizes in a hybrid system of Au-PpIX was studied [205]. Although the LSPR peaks of AuNPs with different sizes (19, 66 and 106 nm) were very close and all have similar extend of spectral overlap with the absorbance of PpIX, the bigger AuNPs would have more plasmonic enhancement in both fluorescence and SOG. The experimental results were further corroborated by the finite-difference time-domain (FDTD) simulations showing that bigger AuNPs could generate a stronger localized electric field due to their stronger scattering as compared to the smaller Au NPs. Our group has obtained similar results for silver nanoparticles as the plasmonic enhancer for AIE-based photosensitizers, where larger AgNPs resulted in a higher ME-SOG than the smaller AgNPs in the studied AgNP@AIE-photosensitizer hybrid systems [20, 22]. Hence, based on the results for both Ag and Au NPs, it could be concluded that bigger metal NPs with stronger scattering and electric field under the incident light would lead to stronger plasmonic enhancement in ROS generation by the PS molecules in the colloidal metal-photosensitizer system.

The shape of metal nanostructures is another key factor in affecting ME-SOG as it determines the enhanced electric field as well as the amount of energy transfer from the excited PS molecule to the metal nanostructure at any individual incident light with certain wavelength. For example, Mthethwa and Nyokong [196] have shown that Au nano-bipyramids and Au nanorods (NRs) could achieve comparable SOG enhancement for AlPc (2-fold for the Au bipyramids and 1.96-fold for AuNRs). Despite that AuNRs have larger spectral overlap with the AlPc molecules, which supposed to have better enhancement, this result suggested that the role of asymmetric shape and anisotropicity on the enhanced electric field around metal nanostructure in ME-SOG is non-trivial [210]. Other studies have also shown that Au nanorods have better ME-SOG performance as compared with spherical AuNPs when the colloidal metal-PS system is irradiated by visible light [252]. However, the opposite trend was observed by Hayden et al. [214], when PpIX was attached to the spherical AuNPs and AuNRs, respectively (i.e., AuNRs has lesser SOG enhancement effect than the spherical AuNPs). Recently, Macia et al. [224] reported a 4-fold enhancement in SOG of Rose Bengal by using silver nanocubes (edge length of 46 nm) as the plasmonic enhancer, which enhancement is higher than that of using spherical AgNPs [223] in the same system and other reported value for RB in the colloidal metal-PS system. They concluded that the higher SOG enhancement factor is due to the enhanced electric field at sharp corners and edges of Ag nanocube. Furthermore, the shape of metal NPs affect the two-photon singlet oxygen generation. It was observed that the Au bipyramid led to more than 11-fold enhancement in two-photon SOG by the sulfonated-AlPc while Au nanorod only resulted in about 2-fold enhancement [219].

##### Illumination Wavelength for Singlet Oxygen Generation

As the electric field around the plasmonic NPs is dependent on the wavelength of incident light, another important parameter for ME-SOG is the wavelength of incident light. It should be mentioned that although the electric field reaches a maximum when an individual nanostructure is irradiated by light with the same wavelength of LSPR peak (*λ*_max_), the enhanced electric field would not be significant for the case of using light irradiation with longer wavelengths. Hayden et al. [214] have shown that spherical AuNPs enhanced the SOG at about 13-fold when the colloidal metal-PpIX system is irradiated by a red light, while AgNPs were not able to enhance the SOG. These results are consistent with the theoretical background of ME-SOG. Photosensitizer with multi-wavelength excitation is a good candidate to study the effect of excitation wavelength. Hence, Kotkowiak and Dudkowiak [212] have studied the effect of excitation wavelength on ME-SOG enhancement of two different photosensitizers (i.e., pheophorbide and hematoporphyrin) in the presence of AuNPs. It was observed that stronger SOG enhancement was obtained when the photosensitizer molecules were excited by the incident light closer to its *λ*_max_.

##### The Distance Between Metal Nanoparticles and Photosensitizer Molecule

As shown previously, metal-enhanced ROS could occur even with direct attachment of PS molecule onto the surface of metal NPs. It should be mentioned that “direct attachment” here refer to the attachment in presence of the capping agent on metal NPs, which isolates the photosensitizer molecule and metal NPs without additional spacer. Although theoretical studies have shown that the electric field is maximum at the surface of metal NPs, which decreases exponentially by increasing the distance from the surface, there is no general trend observed for the distance-dependency of the ME-SOG in colloidal systems. Several studies have shown the ME-SOG by direct conjugation of photosensitizer molecules to the metal NPs in the presence of capping agent between them via electrostatic or covalent binding [193, 197, 199, 200, 214]. Hayden et al. [214] reported that the use of a shorter PEG as capping agent of metal NPs is more favorable for ME-SOG than the long-chain PEG. Other than direct attachment, dielectric spacers such as silica can be used to control the distance between the photosensitizer molecule and metal NP. For example, Lismont et al. [248] have developed the Ag@Silica@photosensitizer system by varying the spacer thickness. The maximum SOG was found in the Ag@Silica@photosensitizer with 5 nm thick spacer while SOG efficiency decreased with the increase in the silica shell thickness [248]. On the other hand, Planas et al. [223] found that SOG quenched at a very short distance and reached a maximum value at around 10 nm shell thickness in the Ag@silica@Rose Bengal colloidal solution, and SOG efficiency decreases when silica thickness is larger 10 nm. This “bell curve” like distance-dependent ME-SOG behavior of PS molecules could be due to the contribution of different factors, which include the (1) enhanced excitation rate due to the electric field, (2) energy transfer from the excited PS to metal NP via nanometal surface energy transfer (NSET) or Förster resonance energy transfer (FRET) mechanisms, (3) competition between intersystem crossing, radiative and non-radiative pathways, and (4) efficiency of different triplet–triplet annihilation processes. In our recent study, we have investigated the distance-dependent behavior of ME-SOG and its correlation with MEF in the same metal-PS colloidal system. In this work, a layer-by-layer approach is used to tune the thickness of the dielectric layer in a few nanometer scale (2–3 nm) [20]. It was observed that ME-SOG requires a shorter distance than the MEF, which could be attributed to the non-radiative nature of this phenomenon.

##### Molar Ratio of Metal Nanoparticles to Photosensitizer

**Fig. 15:**
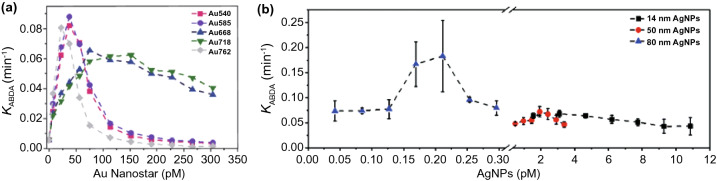
Plot of kinetic constant based on degradation of ABDA (kABDA) versus different concentration of metal nanoparticles showing the plasmonic enhancement effect on the singlet oxygen generation (SOG) efficiency of **a** AIE-PS nanodots (2 μg mL^−1^) on Au nanostars with different maximum extinction peaks, reproduced from Ref. [21] with permission from Nature-Springer. **b** Red-emissive AIE-PS molecule on AgNPs, reproduced from Ref. [22] with permission from Royal Society of Chemistry

The metal NPs-to-photosensitizer molecule molar ratio (M-P) might also affect ME-SOG. Some studies suggested that the SOG can be enhanced by increasing the concentration of metal NPs to the solution of photosensitizer molecules in forming the metal-PS colloidal system [209, 230, 265]. The increase in metal NPs concentration can enhance electric field as a result of higher surface coverage of photosensitizer molecules. However, Yu et al.[250] have observed that the SOG enhancement factor was much higher when a lower amount of methylene blue was loaded to the Au@silica NPs. Therefore, it seems that there might be a critical M-P ratio to achieve the maximum ME-SOG. Recently, we have studied the effect of concentration of metal nanoparticles (i.e., spherical AgNPs and anisotropic Au nanostars) on ME-SOG of different AIE-PS dots [21, 22]. In both studies, we found that there is an optimum M-P ratio for maximum ME-SOG, when the surfaces of metal nanoparticles are fully covered with PS molecules (Fig. 15). However, since the curvature of the surface affects the adsorption of the PS molecules, the optimum M-P ratio cannot be predicted based on total surface area and it should be found experimentally for each type of metal nanoparticles with different morphology (i.e., size and shape).

### Beyond Singlet Oxygen Generation: More In-depth Considerations of the Metal-Photosensitizer Systems

The presence of plasmonic nanoparticles in the vicinity of photosensitizer molecules not only affects the SOG generation rate but also have other consequences that provide more in-depth knowledge of the metal-photosensitizer system. The increase in non-radiative decay rates in the vicinity of metal surface is one of the most observed phenomena in the metal-photosensitizer system. The increase in non-radiative decay rates that is due to energy transfer from the excited photosensitizer molecules to the metal surface often led to an increase in the total decay rate, and thus decrease in the fluorescence lifetime [200, 209, 238]. In addition, radiative decay rates can be enhanced due to the enhancement in excitation rate as a result of the enhanced electric field around the metal NPs. A similar effect has been observed in MEF as well. In addition to the decrease in the fluorescence lifetime, the photostability and photobleaching of photosensitizer molecules can be improved as reported in the literature [250]. Fluorescence quenching has been mostly observed for the MEF systems where metal NPs and photosensitizer molecules have direct or close contact (i.e., distance between them is only a few nanometers). Thus, simultaneous enhancement of fluorescence and SOG have been rarely reported. To achieve both MEF and ME-SOG in the same metal-spacer-PS systems, sufficient distance between the PS and metal NPs is required as there is a competition between enhanced excitation and energy transfer, which can be tuned by using a dielectric spacer.

**Fig. 16:**
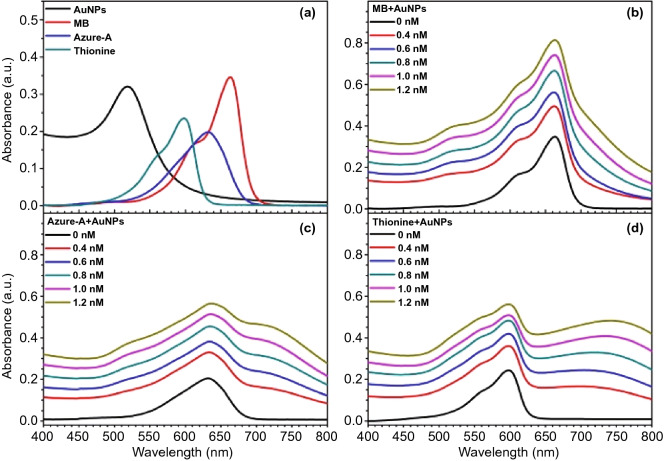
**a** Absorbance spectra of AuNPs and three different photosensitizers including Methylene blue, Azure-A, and Thionine. Absorbance spectra of **b** Methylene blue (5 µM), **c** Azure-A (5 µM), and **d** Thionin (5 µM), in the presence of Au NPs (0.6 nM). The absorbance of Au NPs has been subtracted as background, reproduced from Ref. [209] with permission from American Chemical Society

Besides, the most important factors affecting the performance of the metal-PS system are the enhanced excitation rate and the enhanced absorbance of photosensitizer molecules, which could lead to more efficient ISC and consequently, more SOG generation. Figure 16 shows the enhancement in the absorbance spectra of different photosensitizers in the presence of various amount of AuNPs [209]. In addition to absorbance, the excitation rate can be altered in the presence of metal NPs due to both the enhanced absorbance and enhanced electric field around the NPs. The enhanced in ISC has been observed by transient absorption spectra study [265, 266].

### Metal-Enhanced Singlet Oxygen with Near Infrared Excitation

Until now, most of the developed ME-SOG systems are based on one-photon excitation, where the excitation wavelength is in the visible to near infrared region. However, efficient in vivo PDT cannot be achieved by one-photon excitation due to the high absorption of light by tissue. Therefore, developing ME-SOG systems with excitation wavelength in the near-infrared red region is of importance, as body tissue absorbance is minimum in this region. Generally, the excitation in NIR region can be obtained by incorporation of photosensitizers that can be excited by two-photon light, and with use of upconversion photosensitizers. Two-photon PDT has many advantages over one-photon PDT. (1) Two-photons of infrared light can be used instead of one-photon light with visible wavelength, (2) Two-photon infrared light has less light scattering, and (3) more tissue penetration depth with less absorption by the tissue as compared with the visible light. However, not all of the photosensitizers can be activated by a two-photon laser, which is the current challenge for in vivo applications [267].

As excitation wavelength in two-photon PDT is in the NIR and infrared region, plasmonic nanoparticles with extinction peak located in this region are required for two-photon ME-SOG. Anisotropic Au nanostructures such as nanorods are good candidates for this purpose, owing to their tunable plasmonic peak from near infrared to the infrared region as a function of their aspect ratio [268–273]. There are a few reports on two-photon ME-SOG using the anisotropic Au nanostructures with varied aspect ratios. Chu et al. [218] have synthesized AlPcS linked to Au nanoring for combined photothermal therapy and PDT with 2.9-fold singlet oxygen generation under two-photon excitation. In another study, Au nanorods with silica shell embedding Pd-meso-tetra(4-carboxyphenyl) porphyrin as a photosensitizer were studied for two-photon activated PDT, where 4-fold enhancement of singlet oxygen generation was observed in this system [242]. Zhao et al. [238] have also observed that the AuNR@Silica could enhance the two-photon activated SOG of photosensitizer T790 but not the one-photon SOG. On the other hand, reduction of SOG under two-photon excitation was found in the Au nanocage@lipid layer@hypocrellin system. This could be attributed to the energy transfer from the PS molecule to Au nanocage as well as the sensitivity of two-photon photosensitizer to the UV–Vis light [274].

**Fig. 17:**
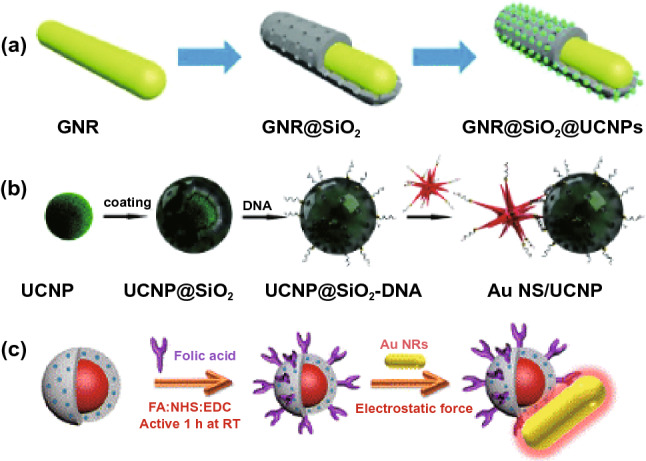
A different approach to fabricate the hybrid system based on plasmonic NPs, PS molecules and UCNPs interactions: **a** formation of core/satellite shape, reproduced from Ref. [255] with permission from Royal Society of Chemistry, **b** DNA hybridization, reproduced from Ref. [259] with permission from Royal Society of Chemistry, and **c** electrostatic attraction forces, reproduced from Ref. [241] with permission from American Chemical Society

Another approach to overcome the penetration depth and absorption of light by tissue is the development of upconversion photosensitizers. Upconversion nanoparticles (UCNPs), e.g., Lanthanide-doped UCNPs can absorb light at a longer wavelength and emit the light with a shorter wavelength [275]. Hence, the upconversion process is of interest due to its nonlinear optical behavior, long excitation wavelength, deep tissue penetration and low autofluorescence background [276, 277]. Similar to the two-photon PDT, plasmonic nanostructures with NIR/IR plasmonic peak can be used for metal-enhanced ROS via upconversion process. Despite many reports on the metal-enhanced upconversion fluorescence [278, 279], there are only a few reports on using UCNPs as an excitation source of other photosensitizers in the visible range in conjunction with NIR/IR plasmonic nanostructures to achieve enhanced ROS [241, 255, 257, 259]. In these systems, the fluorescence intensity of UCNPs can be enhanced due to MEF effect, while the enhanced fluorescence can be used as the excitation source for nearby photosensitizer molecules via FRET process (Fig. 17a). For example, He et al. [259] have used NaYF4:Yb/Er@silica NPs as donor and ZnPc as photosensitizer/acceptor coupled with DNA-functionalized Au nanostars to link with the DNA-functionalized UCNPs@silica. The SOG rate was enhanced at about 2-fold which has been applied for simultaneous PTT and PDT of breast cancer cells (MCF-7) under irradiation of 980 nm laser (Fig. 17b). In another study, a similar hybrid system has been developed based on Au nanorods, methylene blue, and UCNPs via electrostatic interaction between the AuNRs and folic-acid capped UCNPs@silica:Methylene Blue, where a 1.16-fold SOG enhancement was observed [241]. As shown in Fig. 17c, the AuNRs@silica:ZnPc where surrounded by UCNPs via electrostatic interaction. The observed SOG enhancement was about 6–7-fold as compared to the free ZnPc, indicating the role of plasmonic Au NRs and UCNPs in this ME-SOG system. The presence of UCNPs in the system can excite more photosensitizers and consequently, enhance the PDT efficiency.

## Conclusion and Perspectives

This review article summarizes the recent progress in developing new generation of nanomaterial-based photosensitizers, including AIE-photosensitizer nanodots, metal-nanoclusters and carbon dots-based photosensitizer. For AIE-photosensitizer nanodots, there are different ways that can be used to improve the singlet oxygen generation rate by choosing the right type of AIE skeleton, introducing electron donor/acceptor groups, controlling the strength of the electron donor/acceptor groups and the distance between them. For the metal NCs-based photosensitizer, the roles of metal type (e.g., Au, Ag or Au–Ag Alloy), capping agent and number of atoms to affect the rate of ISC has been discussed. Different types of carbon dots-based photosensitizers have been designed and applied for various biomedical purposes, e.g., image-guided photodynamic therapy and photothermal therapy. Despite the unique properties of these nano-photosensitizers, they still suffer from the excitation range limitation as most of them have the excitation wavelength of less than 550 nm. However, this drawback might be resolved using upconversion nanoparticles (e.g., NaYF_4_:Yb/Er) and/or multi-photon excitation.

Other than developing new photosensitizers, plasmonic engineering strategies can be used to enhance the SOG efficiency of photosensitizers. This approach is of interest due to its flexibility to apply for any type of photosensitizes, which relies on the interactions of light with the plasmonic metal nanoparticles (e.g., Au/AgNPs) and photosensitizer that are placed in close proximity. The as-developed metal-enhanced photosensitizers often show higher photostability and minimum photobleaching, rendering it an excellent theranostic agent for image-guided therapy. It has been proven that metal-enhanced singlet oxygen generation is a size-dependent and distance-dependent phenomenon. In addition, different factors such as shape and composition of metal core, excitation wavelength and even metal NPs-to-photosensitizer molecule molar ratio would affect the final SOG enhancement factor. Due to nonlinearity of the system, it is suggested that the optimized conditions could be obtained through systematic studies for each photosensitizer molecule. Thus, the future work would be directed to determine the effective parameters of metal-enhanced SOG as well as seek clarification on some of the inconsistencies reported in different studies. Although there are some similarities between the metal-enhanced fluorescence and metal-enhanced SOG regarding the enhanced excitation rate in the vicinity of metal nanostructures, different observations have been reported by different research groups, which make the understanding of the effective parameters essential. Next step will be to develop some general strategies and design effective plasmonic nanostructures to enhance the ROS generation rate and fluorescence brightness simultaneously for more advance treatment like image-guided PDT.

Overcoming the inconsistencies in measuring the singlet oxygen enhancement factor will be the key to the success of future metal-enhanced SOG system. For example, Karolin and Geddes [167] have shown experimentally that the ROS enhancement factor in metal-enhanced SOG system is a function of light power and that the light with higher power would lead to more ROS enhancement due to the volumetric effect of plasmonic nanostructures. However, there is not yet an established protocol on setting the standard light intensity to measure the ROS enhancement factor for any plasmonic-photosensitizer system. Moreover, the plasmonic nanoparticles show different responses under the incident light of different wavelength. This wavelength-dependent behavior is important as most of the practical PDTs are being done under red to near-infrared (NIR) lasers due to more tissue penetration depth in these regions. Hence, the enhancement factor reported for similar systems, but different conditions are not comparable and might not be observed in the practical PDT. Another inconsistency in the reported data comes from the method to determine the generated singlet oxygen molecules. In addition to spectroscopic measurement of singlet oxygen molecules (i.e., phosphorescence of singlet oxygen at 1270 nm), there are different chemical probes that could be used for indirect monitoring of the singlet oxygen [280]. However, different methods including direct or indirect measurement for the as-generated singlet oxygen might show different responses, leading incomparable results of the ME-SOG enhancement factors obtained from different studies. Hence, developing a systematic quantification and measurement methods for ME-SOG enhancement factor that are applicable to different metal-PS studies is required.

In conclusion, the future of efficient photodynamic therapy is tied to the development of new photosensitizers with good biocompatibility, high efficacy at low concentration or even aggregated state. In particular, successful development of new Type II nano-photosensitizers are of great importance since they can produce singlet oxygen through a fast and direct energy transfer process, which does not need any secondary biological substrate. These new photosensitizers are often enriched with a variety of functional groups which allow for further formulations into nanoparticle, modification with specific biomolecules such as antibody or aptamer as targeting agents, as well as incorporation of plasmonic nanostructures at suitable distance that can be used to enhance the therapeutic effects. It is predicted that the future development of nano-photosensitizers will be tied to the seamless integration of multifunctionalities that combine bioimaging (fluorescent enhancement) and phototherapy (ROS enhancement) for theranostic applications, in addition to exhibit good biocompatibility, high efficacy and low concentration/dosage requirement for effective treatment, even at the aggregated state without affecting their PDT performance. Theoretical calculations and computer simulations can be utilized to reduce the extensive experimental work in metal-enhanced systems and find the best parameters (e.g., size, shape, and distance) leading to the maximum metal-enhanced effect. However, establishing a general protocol on how to measure different ROS generation and determining the enhancement factor seems to be critical for us to have a fair comparison among the different results obtained from different studies. We believe that new advancement in nanobiotechnology and theoretical simulation could accelerate the practical applications of ME-SOG systems toward highly effective theranostic treatment such as image-guided photodynamic therapy in future nanomedicine.
